# Mono- and dinuclear thiophene-based palladium(ii) complexes bearing phosphorus ylides: synthesis, catalytic activity, cytotoxicity and molecular docking studies

**DOI:** 10.1039/d6ra06822g

**Published:** 2026-09-29

**Authors:** Soraya Sepehrfar, Sepideh Samiee, Elham Hoveizi, Jan Janczak, Vladislav Ignatiev, Paulina Maria Dominiak

**Affiliations:** a Department of Chemistry, Faculty of Science, Shahid Chamran University of Ahvaz Ahvaz Iran s.samiee@scu.ac.ir samiee.sepideh@gmail.com +98 6133337009 +98 6133331042; b Department of Biology, Faculty of Science, Shahid Chamran University of Ahvaz Ahvaz Iran; c Institute of Low Temperature and Structure Research, Polish Academy of Sciences Okólna 2 Str. 50-422 Wroclaw Poland; d Biological and Chemical Research Centre, Faculty of Chemistry, University of Warsaw Poland

## Abstract

Two new thiophene-based palladium(ii) complexes, the mononuclear [Pd(L)_2_(Cl)_2_] (M) and [Pd(L)(µ-Cl)]_2_ (D), were synthesized in good yield (73–90%) by reacting equimolar palladium(ii) salts with the thiophene-derived Schiff base ligand, C_10_H_7_N=C(H)(C_4_H_3_S) (L). The reactivity of complex D with unsymmetrical phosphorus ylides [Ph_2_P(CH_2_)_2_PPh_2_C(H)C(O)C_6_H_4_X] (X = Cl (Y^1^), NO_2_ (Y^2^), OCH_3_ (Y^3^), Br (Y^4^)) was also investigated *via* chloride bridge-splitting reactions, affording a new family of mononuclear thiophene-based Pd(ii) complexes (D^1^–D^4^). L and its Pd(ii) complexes were characterized by elemental analysis, FT-IR, and NMR (^1^H, ^13^C, and ^31^P) spectroscopy. Single-crystal X-ray diffraction confirmed the molecular structures of L and complex M, and DFT calculations were additionally performed to support the structural analysis of L. Complexes D^1^–D^4^ exhibited excellent catalytic activity in Suzuki–Miyaura cross-coupling reactions under aerobic conditions in EtOH. MTT assays revealed cytotoxic activity against human carcinoma cell lines HT-29 and A549, with D^4^ showing the highest cytotoxicity. Molecular docking with SARS-CoV-2 (PDB ID: 6Y2F) and transcription factor STAT3B/DNA (PDB ID: 1BG1) indicated that L, complexes M and D^1^ effectively interact with the active sites *via* stable non-covalent interactions, suggesting their potential as anticancer and anti-COVID-19 agents; D^1^ showed superior inhibitory activity against COVID-19.

## Abstract 2

Two new thiophene-based palladium(ii) complexes, the mononuclear [Pd(L)_2_(Cl)_2_] (M) and [Pd(L)(µ-Cl)]_2_ (D), were synthesized (73–90% yield) by reacting palladium(ii) salts with the thiophene-derived Schiff base ligand, C_10_H_7_NC(H)(C_4_H_3_S) (L).

## Graphical abstract

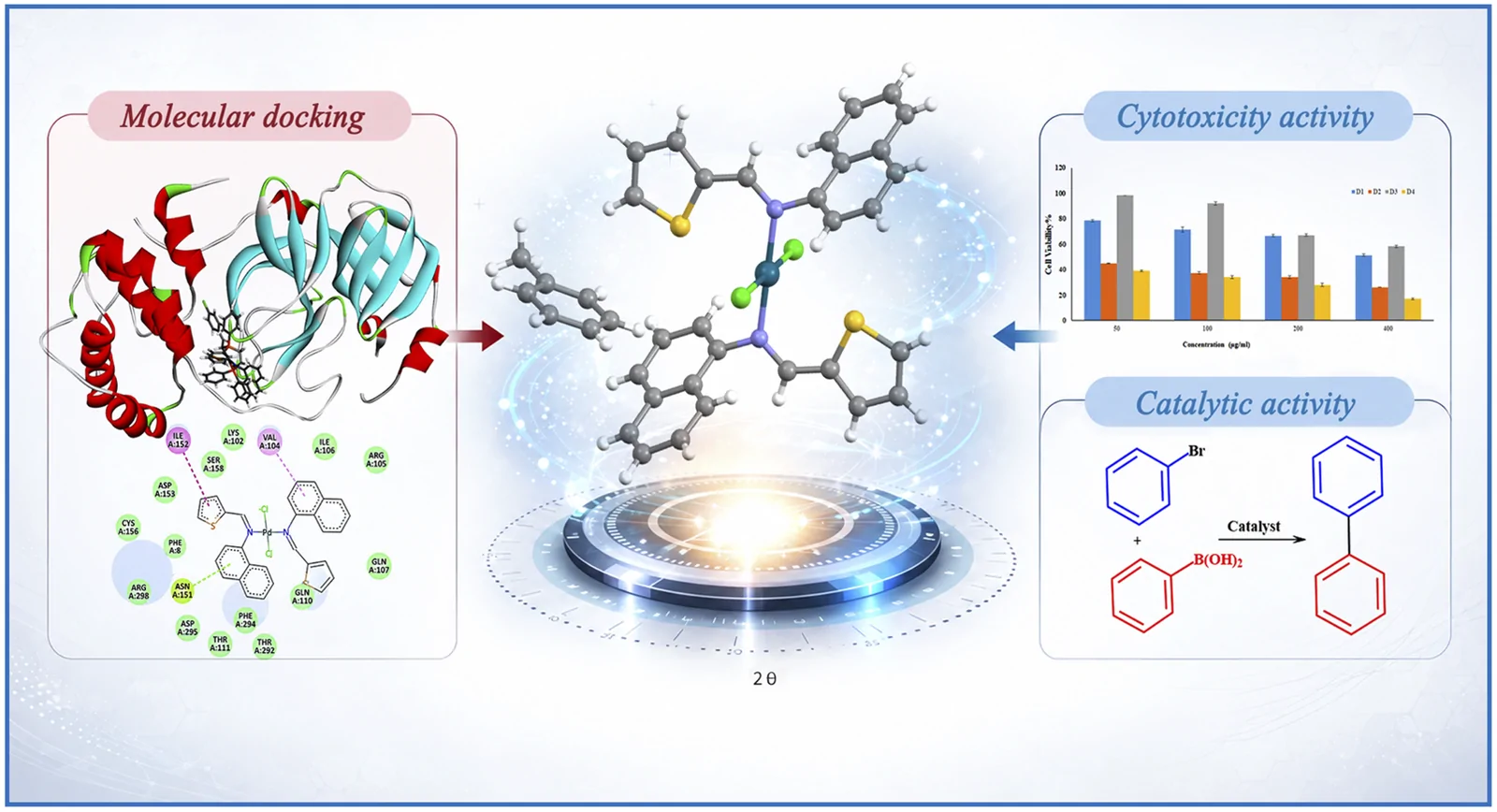

## 1. Introduction

Palladium coordination chemistry has attracted considerable interest in recent years because of its versatile applications in catalysis, supramolecular self-assembly, and medicinal chemistry.^1–4^ As an important platinum-group metal, palladium plays a central role in the development of transition-metal complexes with promising biological properties, including anticancer,^5^ anti-inflammatory,^6^ antibacterial,^7^ and antifungal activities.^8^ Due to limitations of platinum-based chemotherapy, particularly systemic toxicity such as nephrotoxicity and the frequent emergence of drug resistance, palladium(ii) complexes have emerged as promising alternatives for therapeutic applications.^9,10^ Notably, some palladium complexes exhibit distinct mechanisms of action, retain activity against cisplatin-resistant cell lines, and show no mutagenic potential in the Ames test compared with their platinum analogues. Chelating ligands have also been employed to develop Pd(ii) complexes with enhanced stability and biological activity.^9^ For example, Pd(ii) thiosemicarbazone complexes can remain stable in buffered solutions at physiological pH while exhibiting selective cytotoxicity toward tumour cells.^11^ In this regard, the mono- and dinuclear Pd(ii) formamidine complexes have been structurally and theoretically characterized, and evaluated for their cytotoxic activity, providing useful benchmarks for the geometric and electronic parameters of related square-planar Pd(ii) complexes.^12,13^ Thiophene-based palladium complexes are of particular interest because of the inherent electronic, optical, and biological properties associated with the thiophene ring.^14–16^ These complexes have been investigated as efficient catalysts for Suzuki–Miyaura cross-coupling reactions^17,18^ and have also demonstrated antioxidant, antimicrobial, and anticancer activities against human tumour cell lines, in some cases through the induction of apoptosis and disruption of the mitochondrial membrane potential.^19–21^ Despite these advances, the coordination behaviour and biological properties of palladium(ii) complexes incorporating thiophene-containing Schiff-base scaffolds remain relatively underexplored.^22^ Furthermore, the increasing prevalence of drug-resistant pathogens represents a major challenge to effective disease management, reinforcing the need for the development of new therapeutic agents with alternative mechanisms of action.^1^

Schiff-base ligands are of considerable interest because of their convenient synthesis, structural diversity, and tunable coordination properties toward metal centres.^23^ Their applications extend to diverse areas, including photochromism and thermochromism,^24,25^ proton-transfer tautomeric equilibria, biological activity, and the development of biologically active compounds such as antibiotics, anti-inflammatory agents, and antitumour compounds.^26–29^ They have also attracted considerable attention as models for biological systems and because of their antibacterial, antifungal, and anticancer properties.^30–32^ In addition, their versatile coordination behaviour has made them valuable candidates for the development of homogeneous catalysts with potential industrial applications.^33,34^

Phosphorus ylides are particularly intriguing ligands in organometallic chemistry because of their distinctive electronic properties and versatile coordination behaviour.^35^ The α-keto-stabilized ylides derived from diphosphine ligands such as dppm (bis(diphenylphosphino)methane), dppe (1,2-bis(diphenylphosphino)ethane), and dppp (1,3-bis(diphenylphosphino)propane) have attracted increasing interest because of their diverse coordination modes and catalytic applications.^36^ Their ambidentate character allows coordination through the phosphine phosphorus atoms, the ylidic carbon, or the enolate oxygen, giving rise to diverse binding modes, including P, C- and P, O-chelation as well as bridging coordination.^35,37–40^ In this context, the present study focuses on the design, synthesis, structural characterization, and reactivity of mono- and dinuclear thiophene-based palladium(ii) complexes. Furthermore, the reactivity of D toward a series of unsymmetrical diphosphine ylides, [Ph_2_P(CH_2_)_2_PPh_2_C(H)C(O)C_6_H_4_X] (X = Cl (Y^1^), NO_2_ (Y^2^), OCH_3_ (Y^3^), Br (Y^4^)), was investigated. The resulting complexes (D^1^–D^4^) were characterized using elemental analysis, FT-IR, and NMR spectroscopy, while the molecular structures of L and complex M were established by single-crystal X-ray diffraction. In addition, their catalytic in the Suzuki–Miyaura cross-coupling reaction, cytotoxicity toward HT-29 and A549 human carcinoma cell lines, and molecular docking interactions with SARS-CoV-2 Mpro (PDB ID: 6Y2F) and the STAT3β/DNA complex (PDB ID: 1BG1) were also reported.

## 2. Experimental

### 2.1. Materials and characterization

All reactions were performed under a nitrogen atmosphere using standard Schlenk techniques. Reagents and solvents were commercially obtained (reagent grade) and used without further purification. Starting materials, including 2-thiophenecarboxaldehyde, 1-naphthylamine, and palladium(ii) salts, were purchased from Sigma-Aldrich and Merck. Unsymmetrical phosphorus ylides (Y^1^–Y^4^) were synthesized from dppe and the corresponding ketone as previously reported.^41,42^ FT-IR spectra were recorded on a BOMEM MB-Series spectrometer (400–4000 cm^−1^, KBr disk). Elemental analyses were performed using Elmentar Vario CHNS and PerkinElmer instruments. NMR spectra (^1^H, ^31^P{^1^H}, ^13^C{^1^H}) were acquired in CDCl_3_ at 25 °C using Bruker 250 and 400 MHz spectrometers.

### 2.2. Synthesis

#### 2.2.1. Synthesis of thiophene-derived Schiff base ligand (L)

To a stirring ethanolic solution of 1-naphthylamine (0.25 mmol, 0.035 g), 2-thiophenecarboxaldehyde (0.25 mmol, 0.028 g) was added in a 1 : 1 molar ratio (Scheme 1). The mixture was refluxed at 60 °C for 4 hours until a yellow solution formed. Slow evaporation at room temperature yielded yellow crystals after several days. Yield: 85%. Mp 213–214 °C. Anal. Calc. for C_15_H_11_NS (MW = 237.31 g mol^−1^): C, 75.92; H, 4.67; N, 5.90; S, 13.51. Found: C, 75.67; H, 4.65; N, 5.88; S, 13.50%. Selected IR (KBr, cm^−1^): *ν*(C=N) 1635, *ν*(C–S) 859. ^1^H NMR (400 MHz, CDCl_3_) *δ*_H_ (ppm): 8.68 (s, 1H, HC=N), 7.87–8.41 (m, 3H, thiophene ring), 7.09–7.76 (m, 7H, naphthalene ring). ^13^C{^1^H} NMR (60 MHz, CDCl_3_) *δ*_c_ (ppm): 121.12–131.00 (m, Ar-C), 145.71 (s, C–N), 140.29 (C–S), 150.09 (s, HC=N).

**Scheme 1:**
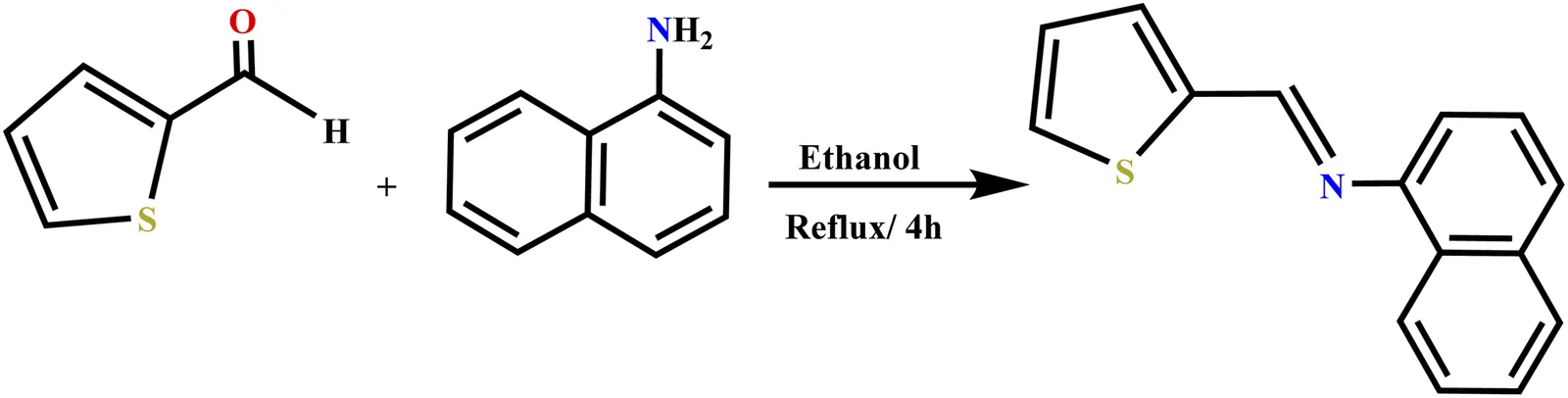
Synthetic route for the preparation of the Schiff base ligand *N*-(2-thienylmethylene) naphthalenamine (L).

#### 2.2.2. Synthesis of mononuclear thiophene-based palladium(ii) complex (M)

In a branched tube, palladium(ii) chloride (0.25 mmol, 0.044 g) and L (0.25 mmol, 0.059 g) were dissolved in toluene. One arm of the tube was heated to 60 °C, while the other remained at room temperature. Over several days, pale yellow cubic crystals of complex M, suitable for X-ray diffraction, formed in the cooler arm. Yield (based on L): 90%. Mp 213–214 °C. Anal. calc. for C_30_H_22_Cl_2_N_2_PdS_2_·2(C_7_H_8_) (MW = 836.18 g mol^−1^): C, 63.20; H, 4.58; N, 3.35; S, 7.67. Found: C, 63.58; H, 4.86; N, 3.17; S, 7.44%. Selected IR (KBr, cm^−1^): *ν*(C=N) 1603, *ν*(C–S) 860. ^1^H NMR (400 MHz, CDCl_3_) *δ*_H_ (ppm): 8.41 (s, 1H, HC=N), 7.60–8.26 (m, 3H, thiophene ring), 7.11–7.56 (m, 7H, naphthalene ring), 2.36 (s, 3H, CH_3_ of toluene). ^13^C{^1^H} NMR (60 MHz, CDCl_3_) *δ*_c_ (ppm): 18.50 (CH_3_ of toluene), 120.03–126.09 (naphthalene-C), 131.66–144.53 (thiophene-C), 163.08 (HC=N).

#### 2.2.3. Synthesis of dinuclear thiophene-based palladium(ii) complex (D)

A solution of palladium(ii) acetate (0.25 mmol, 0.056 g) in 10 mL toluene was mixed with an equimolar solution of L (0.25 mmol, 0.059 g) in 10 mL toluene. This mixture was refluxed at 60 °C for 24 hours under N_2_ with stirring, then filtered through Celite to remove precipitated palladium. The resulting product (0.25 mmol, 0.201 g) was dried under vacuum, dissolved in 15 mL acetone, and treated dropwise with a methanolic LiCl solution (0.8 mmol, 0.034 g) to replace acetate bridges with chlorine (Scheme 2). After 24 hours of stirring, the mixture was filtered to yield complex D. Yield: 73%. Mp 213–214 °C. Anal. calc. for C_30_H_22_Cl_2_N_2_Pd_2_S_2_ (MW = 758.38 g mol^−1^): C, 47.51; H, 2.92; N, 3.69; S, 8.45. Found: C, 47.75; H, 2.82; N, 3.55; S, 8.69%. Selected IR (KBr, cm^−1^): *ν*(C=N) 1610, *ν*(C–S) 768. ^1^H NMR (400 MHz, CDCl_3_) *δ*_H_ (ppm): 8.40 (s, 1H, HC=N), 7.56–8.25 (m, 7H, naphthalene), 7.09–7.45 (m, 3H, thiophene), 2.36 (s, 3H, CH_3_ of toluene). ^13^C{^1^H} NMR (60 MHz, CDCl_3_) *δ*_c_ (ppm): 18.50 (CH_3_ of toluene), 120.03–126.09 (naphthalene-C), 131.66–144.53 (thiophene-C), 163.09 (HC=N).

**Scheme 2:**
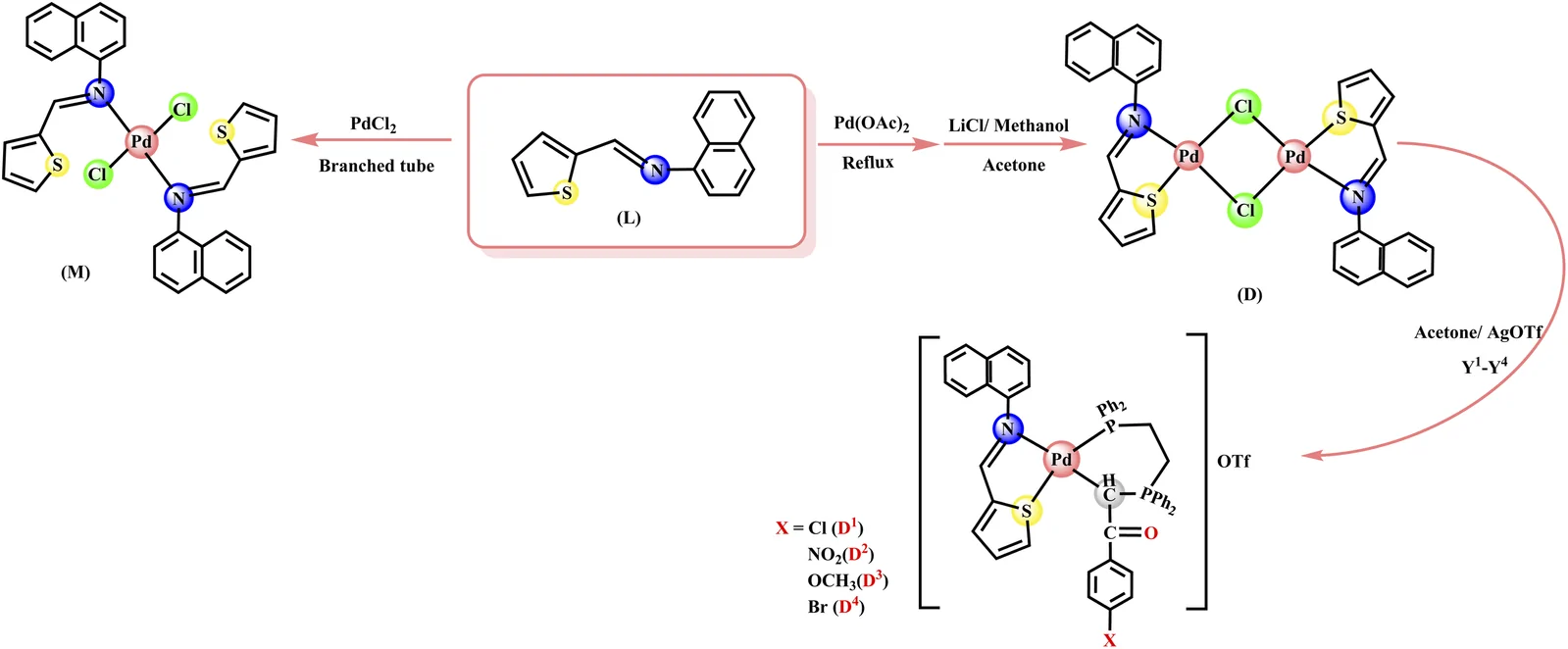
Synthetic strategy for the preparation of thiophene-based palladium(ii) complexes mixed with unsymmetrical phosphorus ylides (note: OTf^−^ is trifluoromethanesulfonate anion, CF_3_SO_3_^−^).

#### 2.2.4. Synthesis of mononuclear thiophene-based palladium(ii) complex bearing diphosphine ylides

To produce complexes D^1^–D^4^, silver trifluoromethanesulfonate (0.029 mmol, 0.007 g) was added to a solution of complex D (0.029 mmol) in 25 mL acetone and stirred for 2 hours. The mixture was then filtered to remove AgCl. Next, each phosphorus ylide (Y^1^–Y^4^) (0.058 mmol) was added and the solution was stirred for 12 hours at room temperature (Scheme 2). The solution was then filtered and concentrated to 2 mL under vacuum. The product was precipitated by adding 10 mL cold *n*-hexane, collected, washed twice with 5 mL cold *n*-hexane, and dried under vacuum.

##### 2.2.4.1. [Pd[C_10_H_7_N=C(H)(C_4_H_3_S)](Y^1^)]OTf (D^1^)

Yield: 79%. Mp 201–203 °C. Anal. calc. for C_50_H_40_ClF_3_NO_4_P_2_PdS_2_ (MW = 1043.81 g mol^−1^): C, 57.53; H, 3.86; N, 1.34; S, 6.14%. Found: C, 57.78; H, 3.95; N, 1.23; S, 5.97.%. Selected IR absorption in KBr (cm^−1^): *ν*(C=O) 1637, *ν*(C=N) 1618, *ν*(OTf^−^) 1251, 1025, *ν*(C–S) 742. ^31^P{^1^H} NMR (CDCl_3_, 101.25 MHz) *δ*_P_ (ppm): 29.18 (d, ^3^*J*_PP_ = 51.53 Hz, PPh_2_), 32.72 (bd, PCH). ^1^H NMR (CDCl_3_, 250.13 MHz) *δ*_H_ (ppm): 2.41–2.86 (m, 2H, CH_2_), 2.94–3.48 (m, 2H, CH_2_), 5.20 (d, ^2^*J*_PH_ = 16.25 Hz, 1H, PCH), 6.90–8.09 (m, 34H, thiophene, naphthalene and phosphine rings), 8.66 (s, 1H, –CH=N). ^13^C{^1^H} NMR (CDCl_3_, 62.90 MHz) *δ*_C_ (ppm): 21.01 (br, CH_2_PPh_2_), 23.49 (br, CH_2_PPh_2_), 36.89 (d, ^1^*J*_PC_ = 122.02 Hz, PCH), 113.03–159.61 (m, Ph), 186.08 (s, C=N), 187.63 (s, C=O).

##### 2.2.4.2. [Pd[C_10_H_7_N=C(H)(C_4_H_3_S)](Y^2^)]OTf (D^2^)

Yield: 74%. Mp 200–202 °C. Anal. Calc. for C_50_H_40_F_3_N_2_O_6_P_2_PdS_2_ (MW = 1054.37 g mol^−1^): C, 56.96; H, 3.82; N, 2.66; S, 6.08%. Found: C, 56.66; H, 3.67; N, 2.60; S, 6.30%. Selected IR absorption in KBr (cm^−1^): *ν*(C=O) 1637, *ν*(C=N) 1618, *ν*(OTf^−^) 1258, 1029, *ν*(C–S) 729. ^31^P{^1^H} NMR (CDCl_3_, 101.25 MHz) *δ*_P_ (ppm): 29.06 (d, ^3^*J*_PP_ = 49.60 Hz, PPh_2_), 32.19 (br, PCH). ^1^H NMR (CDCl_3_, 250.13 MHz) *δ*_H_ (ppm): 2.52–2.62 (m, 2H, CH_2_), 3.06–3.47 (m, 2H, CH_2_), 5.30 (d, ^2^*J*_PH_ = 12.50 Hz, 1H, PCH), 7.11–8.02 (m, 34H, thiophene, naphthalene and phosphine rings), 8.24 (s, 1H, –CH=N). ^13^C{^1^H} NMR (CDCl_3_, 62.90 MHz) *δ*_C_ (ppm): 16.09 (br, CH_2_PPh_2_), 19.27 (br, CH_2_PPh_2_), 115.22–163.66 (m, Ph), 187.99 (s, C=N), 192.45 (s, C=O).

##### 2.2.4.3. [Pd[C_10_H_7_N=C(H)(C_4_H_3_S)](Y^3^)]OTf (D^3^)

Yield: 85%. Mp 203–205 °C. Anal. calc. for C_51_H_43_F_3_NO_5_P_2_PdS_2_ (MW = 1039.39 g mol^−1^): C, 58.93; H, 4.17; N, 1.35; S, 6.17%. Found: C, 58.62; H, 4.36; N, 1.16; S, 6.22%. Selected IR absorption in KBr (cm^−1^): *ν*(C=O) 1636, *ν*(C=N) 1616, *ν*(OTf^−^) 1255, 1033, *ν*(C–S) 735. ^31^P{^1^H} NMR (CDCl_3_, 101.25 MHz) *δ*_P_ (ppm): 29.10 (d, ^3^*J*_PP_ = 51.63 Hz, PPh_2_), 39.28 (br, PCH). ^1^H NMR (CDCl_3_, 250.13 MHz) *δ*_H_ (ppm): 2.28–2.52 (m, 2H, CH_2_), 3.24–3.79 (m, 2H, CH_2_), 3.87 (s, 3H, OCH_3_), 5.14 (d, ^2^*J*_PH_ = 10.25 Hz, 1H, PCH), 6.78–8.08 (m, 34H, thiophene, naphthalene and phosphine rings), 8.68 (s, 1H, –CH=N). ^13^C{^1^H} NMR (CDCl_3_, 62.90 MHz) *δ*_C_ (ppm): 20.74 (br, CH_2_PPh_2_), 23.68 (br, CH_2_PPh_2_), 35.44 (br, PCH), 56.34 (s, CH_3_), 113.86–161.68 (m, Ph), 185.57 (s, C=N), 194.05 (s, C=O).

##### 2.2.4.4. [Pd[C_10_H_7_N=C(H)(C_4_H_3_S)](Y^4^)]OTf (D^4^)

Yield: 73%. Mp 204–206 °C. Anal. calc. for C_50_H_40_BrF_3_NO_4_P_2_PdS_2_ (MW = 1088.26 g mol^−1^): C, 55.18; H, 3.70; N, 1.29; S, 5.89%. Found: C, 55.35; H, 3.68; N, 1.21; S, 5.78%. Selected IR absorption in KBr (cm^−1^): *ν*(C=O) 1636, *ν*(C=N) 1620, *ν*(OTf^−^) 1259, 1030, *ν*(C–S) 742. ^31^P{^1^H} NMR (CDCl_3_, 101.25 MHz) *δ*_P_ (ppm): 29.06 (d, ^3^*J*_PP_ = 49.60 Hz, PPh_2_), 33.21 (br, PCH). ^1^H NMR (CDCl_3_, 250.13 MHz) *δ*_H_ (ppm): 2.28–2.52 (m, 2H, CH_2_), 3.74–3.86 (m, 2H, CH_2_), 5.25 (br, PCH), 6.59–8.09 (m, 34H, thiophene, naphthalene and phosphine rings), 8.57 (s, 1H, –CH=N). ^13^C{^1^H} NMR (CDCl_3_, 62.90 MHz) *δ*_C_ (ppm): 14.15 (br, CH_2_PPh_2_), 23.36 (br, CH_2_PPh_2_), 38.71 (br, PCH), 119.09–141.76 (m, Ph), 166.01 (s, C=N), 191.22 (s, C=O).

### 2.3. X-ray structure solution and refinement

X-ray intensity data for the selected yellow good-quality single crystal of Schiff base ligand (L) were collected using graphite monochromatic MoKα radiation on a four-circle κ geometry Xcalibur diffractometer with Sapphire2 area CCD detector, whereas for the orange crystal of mononuclear complex (M) the diffraction data were collected on an Agilent Technologies SuperNova Dual Source HyPiX 6000HE diffractometer with CuKα radiation (*λ* = 1.54184 Å). Data collections were made using the CrysAlisPro 1.171.42.93a program package.^43^ Integration, scaling of the reflections, correction for Lorenz and polarisation effects and absorption corrections were performed using the CrysAlisPro 1.171.42.93a program package.^43^ Using Olex2,^44^ the structures were solved by direct methods using SHELXT-2014/7 (ref. 45) and refined using SHELXL-2018/3 program.^46^ The hydrogen atoms joined to aromatic carbon atoms were introduced in their geometrical positions and treated as rigid. The final difference Fourier maps showed no peaks of chemical significance. Details of the data-collection parameters, crystallographic data, and final refinement parameters are summarized in Table 1. Visualizations of the structures were made with the Diamond 3.0.^47^ Hirshfeld surface analysis and two-dimensional fingerprint plots for the L crystal were generated using Crystal Explorer 3.1 software.^48^ DFT geometry optimization of L was performed using the Gaussian 09 program^49^ at the B3LYP/6-311++G(d,p) level of theory, starting from the X-ray crystal geometry.

**Table 1:** Crystallographic data and final refinement parameters for crystals of L and complex M

	L	M
Formula	C_15_H_11_NS	C_30_H_22_Cl_2_N_2_PdS_2_·2(C_7_H_8_)
Molecular weight	237.31	836.18
Temperature (K)	295 (2)	100 (2)
Radiation, *λ* (Å)	0.71073	1.54184
Crystal system	Orthorhombic	Triclinic
Space group	*Aba2*	*P*1̄
*a* (Å)	10.7498 (5)	7.8836 (1)
*b* (Å)	21.3221 (10)	9.2826 (1)
*c* (Å)	10.7754 (5)	13.5333 (2)
*α* (°)		94.310 (1)
*β* (°)		98.299 (1)
*γ* (°)		105.565 (1)
*V* (Å^3^)	2469.8 (2)	937.30 (2)
*Z*	8	1
*F*(000)	992	428
*D*_cal_ (g cm^−1^)	1.276	1.481
*θ* range (°)	2.842–29.445	6.648–148.9
*µ* (mm^−1^)	0.237	6.609
Crystal size (mm)	0.287 × 0.276 × 0.077	0.29 × 0.18 × 0.12
*T*_min_/*T*_max_	0.9546/1.000	0.281/0.891
Total/unique/obs. refls (*F*^2^ > 2*σ*(*F*^2^))	8645/2895/2162	17 422/3821/3725
*R*_int_	0.0236	0.0551
*R* [*F*^2^ > 2*σ*(*F*^2^)]^a^	0.0379	*R*_1_ = 0.0300, w*R*_2_ = 0.0789
w*R* [*F*^2^ all refls]^a^	0.0842	*R*_1_ = 0.0310, w*R*_2_ = 0.0795
Flack parameter	−0.03(4)	–
*S*	1.002	1.0445
Δ*ρ*_max_, Δ*ρ*_min_ (e Å^−3^)	+0.109; −0.142	0.43/−0.52

a *R* = Σ‖*F*_o_| − |*F*_c_‖/Σ*F*_o_, w*R* = [Σ[*w*(*F*_o_^2^ − *F*_c_^2^)^2^]/Σ*wF*_o_^4^]^½^; *w*^−1^ = *σ*^2^(*F*_o_^2^) + (*aP*)^2^ + *bP* where *P* = (*F*_o_^2^ + 2*F*_c_^2^)/3. The *a* and *b* parameters are 0.0395 and 0.1471 for L and 0.0329 and 0.9837 for M.

### 2.4. The typical procedure for the Suzuki–Miyaura reaction

The Suzuki–Miyaura cross-coupling reaction was examined using palladium–phosphorus complexes as catalysts, following our previous studies.^18,50^ In a typical experiment, bromobenzene (0.5 mmol, 0.078 g), phenylboronic acid (0.6 mmol, 0.073 g), K_2_CO_3_ (0.75 mmol, 0.104 g), and ethanol (2 mL) were added to a 25 mL round-bottom flask. Complex D^2^ (0.47 mol% Pd) was then added as a solid. The mixture was stirred at 60 °C under atmospheric conditions in a preheated oil bath with a reflux condenser. Reaction progress was monitored by TLC. Upon completion, the catalyst was filtered, and the reaction mixture was extracted with H_2_O and *n*-hexane. The organic layer was dried over anhydrous Na_2_SO_4_ (1.5 g), and *n*-hexane was evaporated to yield pure biphenyl derivatives in 76–98%.

### 2.5. Cytotoxicity assay

#### 2.5.1. Cell culture

A549, HT-29, and fibroblast cells (Pasteur Institute of Iran) were cultured in T75 flasks (SPL, Korea) using DMEM (Gibco, USA) supplemented with 10% FBS (Gibco, USA) and 1% penicillin/streptomycin (Gibco, USA). Cells were maintained at 37 °C in a humidified atmosphere containing 5% CO_2_ and were monitored daily for morphology, confluence, and proliferation under an inverted microscope. The culture medium was replaced every 2–3 days, and cells were subcultured following detachment with trypsin/EDTA (Gibco, USA).

#### 2.5.2. Cell viability assay

A549, HT-29, and fibroblast cells were seeded in 96-well plates at 10 000 cells per well and incubated for 12 h. Cells were then treated with complexes D^1^–D^4^ at concentrations of 50, 100, 200, and 400 µg mL^−1^ and incubated at 37 °C with 5% CO_2_; untreated cells served as controls. An MTT solution (5 mg mL^−1^ in PBS, Sigma, USA) was prepared, diluted 1 : 10 in culture medium, and 100 µL was added to each well. After 4 h, the wells were aspirated, 100 µL of DMSO (Sigma, USA) was added to dissolve the formazan crystals, and the plates were gently shaken for 15 min. Absorbance was measured at 570 nm using an ELISA reader (Fax Stat 2100, USA).

#### 2.5.3. Acridine orange/ethidium bromide staining

Acridine orange/ethidium bromide (AO/EB) staining was used to assess the effect of the IC_50_ dose of complex D^4^ (the most potent complex) on cell viability. A549 and HT-29 cells were seeded at 50 000 cells per well in 24-well plates and exposed to the IC_50_ concentration of D^4^, with untreated cells serving as the control. Cells were stained with a 1 : 1 AO/EB mixture for 3 min, rinsed with PBS, and examined under an inverted microscope (Nikon, Japan).

### 2.6. Molecular docking studies

Computational methods were employed to identify and design novel anticancer and anti-COVID-19 candidates by targeting the corresponding molecular docking receptors. The structures of the target proteins – transcription factor STAT3β/DNA (PDB ID: 1BG1) and the main protease of SARS-CoV-2 (PDB ID: 6Y2F) – were retrieved in PDB format from the RCSB Protein Data Bank (https://www.rcsb.org/structure/, 1BG1 and 6Y2F) to predict the binding modes of L, M, and D^1^ within the active site of Mpro and the SH2 binding site of STAT3β.^51^ Docking was performed using Molegro Virtual Docker software. To create the interaction with the receptor, the process was carried out as follows: First, “hole identification” was performed in five parts of the protein structure. Then, in the interaction process, the internal ES, SP^2^, SP^2^ twists, internal H_bond_, as well as post-binding energy minimization parameters were determined. The outputs were saved in MOL2 format for further visualization and analysis. Next, Molegro Molecular Viewer and Discovery Studio 3.0 software were used to visualize and study the interactions of L, M, and D^1^ with the STAT3/SARS-CoV-2 SH2 binding site.^52–55^

## 3. Results and discussion

### 3.1. Synthesis and characterization

The thiophene-derived Schiff base ligand, *N*-(2-thienylmethylene)-1-naphthalenamine (L), was synthesized by the condensation reaction of 1-naphthylamine and thiophenecarboxaldehyde in ethanol (Scheme 1). The ligand was characterized by FT-IR, ^1^H and ^13^C NMR spectroscopy, and elemental analysis, and its molecular structure was confirmed by single-crystal X-ray diffraction. The reaction of L with palladium(ii) chloride and palladium(ii) acetate in toluene afforded two different products, the yellow mononuclear complex [Pd(L)_2_Cl_2_] (M) and the orange chloride-bridged dinuclear complex [Pd(L)(µ-Cl)]_2_ (D), respectively (Scheme 2). Although both reactions employed a formal 1 : 1 (Pd : L) ratio, single-crystal X-ray diffraction confirmed that, in complex M, the ligand coordinates only through the imine nitrogen, with the thiophene sulfur remaining uncoordinated. This finding can be attributed to two reasons: (1) the weak donor character of thiophene sulfur, whose lone pair conjugation with the aromatic π-system reduces its Lewis basicity and coordination ability, preventing it from effectively competing with the strong, anionic chloride ligands, (2) the synthetic conditions employed for PdCl_2_, which is poorly soluble in toluene and was reacted *via* the branched-tube technique, favoring the bis-ligand, monodentate N-coordinated complex M. Notably, this behaviour is consistent with related thiophene-derived Schiff base Pd(ii) complexes reported in the literature, in which the thiophene sulfur likewise remains uncoordinated.^4,23,56–58^ In contrast, Pd(OAc)_2_ is fully soluble in toluene and reacts homogeneously under reflux, where each Pd center has access to one equivalent of L; the more labile bridging acetate ligand facilitates formation of a dinuclear complex, and subsequent reaction with LiCl replaces the acetate bridges with chloride to give complex D.

Subsequently, the reactivity of complex D toward diphosphine ylides (Y^1^–Y^4^) was investigated. These reactions resulted in chloride-bridge splitting and afforded the mononuclear palladium(ii) complexes (D^1^–D^4^), in which the thiophene-derived Schiff-base (S^N) and phosphorus ylide (P^C) ligands coordinate to the Pd(ii) center. The synthesized complexes were stable in air and moisture. All complexes were characterized by ^1^H, ^31^P, and ^13^C NMR, IR spectroscopy, and elemental analysis. The analytical data were consistent with the proposed formulations, as summarized in Table 2.

**Table 2:** Spectroscopic data for complexes D^1^–D^4^

	IR	^1^H NMR	^31^P NMR		^13^C NMR	Ref.
	*ν*(CO)	*δ*(PCH)	*δ*(PPh_2_)	*δ*(PCH)	*δ*(CO)	
Y^1^	1508	4.17 (br)	−15.13 (46.1)	14.58 (46.4)	183.91	41
D^1^	1638	5.20 (16.2)	29.18 (51.53)	32.72 (br)	187.63	This work
Y^2^	1531	4.23 (br)	−15.22 (48.1)	14.71 (46.8)	182.37	41 and 42]
D^2^	1637	5.30 (12.5)	29.06 (49.60)	32.19 (br)	192.45	This work
Y^3^	1488	4.18 (d)	−15.15 (47.5)	14.33(47.4)	185.04	41
D^3^	1636	5.14 (10.2)	29.10 (51.63)	39.28 (br)	194.05	This work
Y^4^	1517	4.18 (br)	−15.15 (46.3)	14.55 (48.2)	183.95	41
D^4^	1637	5.25 (br)	29.06 (49.60)	33.21 (br)	191.22	This work

The IR spectrum of the free ligand (L) showed a strong azomethine *ν*(C=N)_imine_ peak at 1635 cm^−1^, consistent with a Schiff-base ligand. The thiophene ring (C–S–C) absorption band appeared at 859 cm^−1^. The disappearance of the 2-thiophenecarboxaldehyde *ν*(C=O) band confirmed complete reaction and successful imine ligand synthesis. The ^1^H NMR spectrum of L displayed a singlet at *δ* 8.68 ppm, assigned to the –CH=N– moiety. The multiplets in the 7.09–8.41 ppm range are attributable to the aromatic ring hydrogens of thiophene and naphthalene. In the ^13^C NMR spectrum, the azomethine resonance at *δ* 150.09 ppm replaced the carbonyl resonance of the thiophene moiety, further indicating its disappearance. The aromatic rings related to imine and thiophen and appeared in the 121.12–145.71 ppm range.^20,23,59–61^

It is well documented that the IR spectroscopy identifies functional groups and coordination sites using frequencies. In the spectra of Pd(L)_2_(Cl)_2_ (M) and Pd(L)(µ-Cl)_2_ (D) complexes, the *ν*(C=N)_imine_ shifted to a lower wavenumbers (≈1600 cm^−1^) in comparison to the free ligand, indicating the imine nitrogen is involved in bonding.^62,63^ In complex M, the *ν*(C–S) vibration at 860 cm^−1^, similar to free L (859 cm^−1^), indicates non-coordination of the thiophene sulfur. Conversely, in complex D, a significant shift of this band to a lower frequency (768 cm^−1^) suggests coordination thought thiophene sulfur.^23,63–65^ In the ^1^HNMR, the HC=N proton resonance shifted to a lower frequency (≈*δ* 8.4 ppm) in both complexes, confirming coordination through the lone pair of nitrogen atom.^62,66,67^ The low-field shift of the CH=N carbon signals in the ^13^C{^1^H} NMR spectrum (≈*δ* 163 ppm) confirms azomethine nitrogen coordination to the palladium metal center.^62,68^

The mononuclear complexes (D^1^–D^4^) were successfully characterized by IR and NMR spectroscopy, and their formulations were confirmed by analytical data. Consistent with literature reports, coordination of phosphorus ylides *via* carbon leads to an increase in *ν*(C=O), while oxygen coordination leads to a decrease.^36^ The IR spectra of complexes D^1^–D^4^ exhibit *ν*(C=O) bands in the range of 1636–1638 cm^−1^, showing a significant increase compared to the free ylides (Y^1^–Y^4^).

The ^31^P{^1^H} NMR spectra of complexes D^1^–D^4^ exhibited two doublets at approximately 29 and 38 ppm, corresponding to the PCH and PPh_2_ groups, respectively. These signals were significantly downfield-shifted compared to the free phosphorus ylide (Table 2), indicating ylides (Y^1^–Y^4^) coordination to metal atom through phosphonium and phosphine groups, consistent with previous reports for similar P, C-chelated palladium complexes.^41,42,69^ In the ^1^H NMR spectra of thiophene-based complexes D^1^–D^4^, the methine proton (PCH) signals (typically 5–6 ppm) shift significantly compared to free phosphorus ylides, indicating ylide coordination *via* the ylidic carbon (Table 2). The aromatic protons resonate between 6–8 ppm, while peaks at 2–3.5 ppm correspond to the PCH_2_CH_2_P groups of the ylide ligands in all complexes. A sharp singlet at 3.87 ppm in complex D^3^ confirms the presence of a methoxy (OCH_3_) group. It is noteworthy that the azomethine group resonance appears in the same region for both mono- and dinuclear complexes (8.2–8.6 ppm). The ^13^C{^1^H} NMR spectra of the mononuclear complexes D^1^–D^4^ provide clear evidence regarding the coordination environment of the phosphorus ylide ligands. The ^13^C NMR spectra revealed ylidic carbon resonances at *δ*_C_ 36.89, 35.44, and 38.71 ppm for D^1^, D^3^, and D^4^, respectively (Table 2). A notable feature of the ^13^C NMR spectra of these complexes is the upfield shift of the ylidic carbon signal relative to the free ylides (*δ*_C_ 46–50 ppm),^41,42^ consistent with coordination of the phosphorus ylide through the ylidic carbon (C_α_). This enhanced shielding, attributed to the change in hybridization of the ylidic carbon upon coordination to the metal centre, has also been observed for other C-coordinated ylide complexes.^40,70–72^ Notably, the ylidic carbon signal for D^2^ could not be unambiguously assigned, most likely owing to line broadening;^73^ similar behaviour has been observed for related phosphorus ylide complexes^74^ and for our previously reported complexes.^40,41,75^ In addition, the carbonyl (C=O) resonances appear in the range of 187–194 ppm, showing a significant downfield shift compared to those observed for the corresponding free phosphorus ylides (Table 2). Also, resonances attributed to aromatic carbon atoms are detected within the *δ* 114–167 ppm region, which was consistent with a complex format.^16,40,50,57,74^ Thus, spectroscopic data confirm that unsymmetrical phosphorus ylides Y^1^–Y^4^ coordinate to the Pd(ii) center *via* both the ylidic carbon (PCH) and phosphine (PPh_2_) moieties.

### 3.2. Description of crystal structures

#### 3.2.1. Molecular structure of [C_10_H_7_N=C(H)(C_4_H_3_S)] (L)

This ligand crystallizes in the non-centrosymmetric space group *Aba2* of the orthorhombic system with eight molecules per unit cell. The asymmetric L unit contains only one molecule as shown in Fig. 1a, while the DFT-optimized L structure is shown in Fig. 1b.

**Fig. 1:**
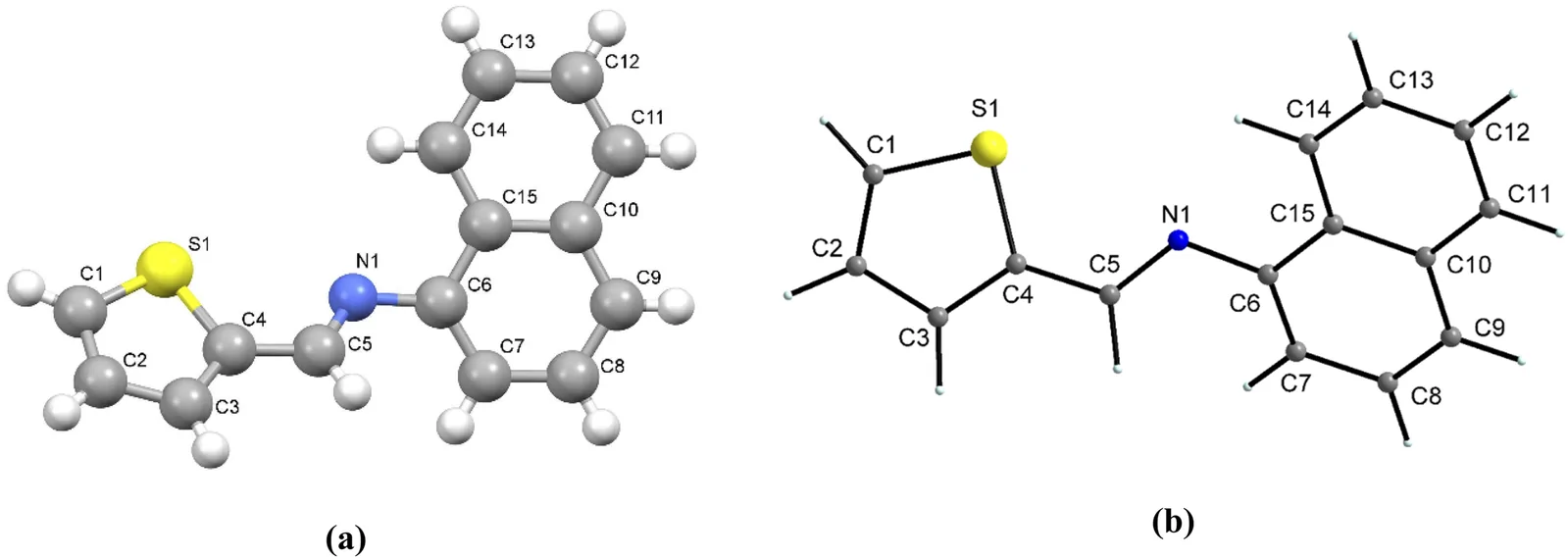
(a) X-ray and (b) DFT optimized structure of the thiophene-derived Schiff base ligand (L) at the B3LYP/6-311++G(d,p) level of theory.

Selected geometric parameters are given in Table 3 along with the DFT calculated values. The full list of the DFT parameters is listed in Table S1 in SI. The conformation of the entire L molecule is not planar, but both fragments of the molecule attached to the central nitrogen atom (N1) are planar, and the average deviation of the atoms from the plane is 0.012 Å and 0.025 Å for the S1, C1–C5, N1 and the N1, C6–C15 fragments, respectively. The dihedral angle between the planar fragments is 33.1(4)°.

**Table 3:** Selected geometric parameters of L obtained from single-crystal X-ray diffraction and DFT calculations at the B3LYP/6-311++G(d,p) level of theory

	X-ray	DFT	L in complex M
**Bond lengths (Å)**			
S1–C1	1.713(3)	1.729	1.709(3)
S1–C4	1.720(3)	1.747	1.731(2)
N1–C5	1.276(3)	1.279	1.293(3)
N1–C6	1.408(3)	1.400	1.443(3)

**Bond angles (°)**			
C1–S1–C4	91.2(2)	91.12	91.15(13)
C5–N1–C6	118.8(2)	120.43	116.6(2)

**Torsion angles (°)**			
S1–C4–C5–N1	−5.9(4)	1.89	0.4(4)
C4–C5–N1–C6	178.1(2)	−175.79	176.9(2)
C5–N1–C6–C15	−144.2(2)	−141.03	−61.4(3)
N1–C6–C15–C14	−2.8(4)	1.97	−7.2(3)

In the gas phase, a similar non-planar conformation of the whole L molecule is observed, as shown by DFT calculations, however, the dihedral angle between the planar fragments connected to N1 is much larger and amounts to 44.60°. Looking at these results, the slight differences between the conformation of the whole molecule obtained by X-rays and DFT studies are due to the different approach of the two methods: X-rays describe the conformation of the molecule in a crystal, in which the intermolecular interactions leading to nucleation and crystal growth influence the conformation of the molecule, whereas in DFT calculations for a single molecule in the gaseous state these interactions are neglected, hence the differences.

Typical directional interactions, such as hydrogen bonds, between L molecules are not observed in the crystal. The packing pattern of L molecules in the crystal, viewed along the *b*-axis, is shown in Fig. 2.

**Fig. 2:**
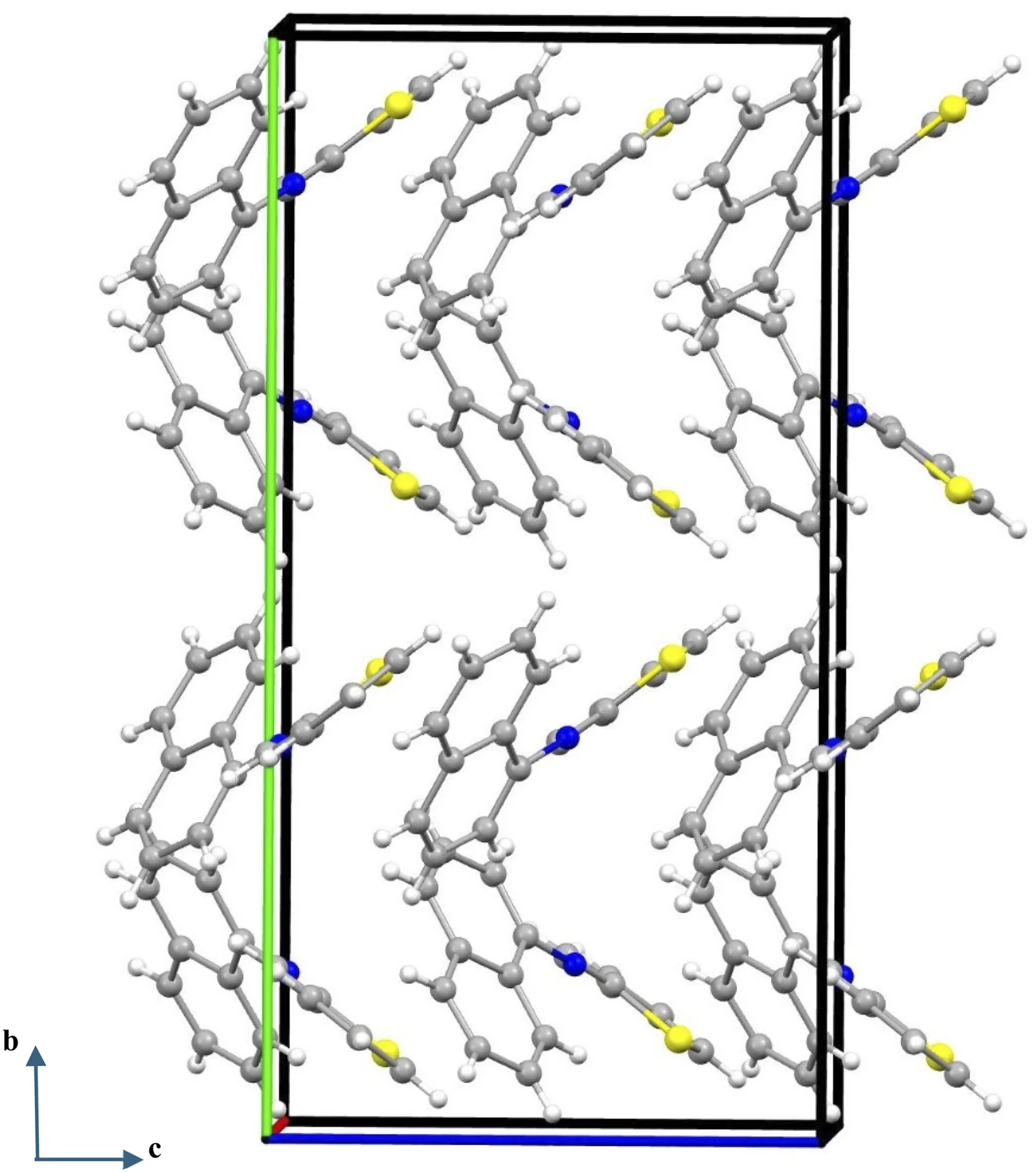
Arrangement of L molecules in the crystal viewed along *b*-axis.

The main interactions between L molecules, as shown by Hirshfeld surface (HS) analysis and 2DFP plots (Fig. S1, SI), are dispersion forces and weak C–H⋯C, S⋯H, and C–H⋯N interactions, which constitute 40.3%, 40.4%, 11.6%, and 5.0%, respectively, which is 97.3% of the HS contribution. S⋯C/C⋯S (1.7%) and C⋯C (1.0%) interactions complete the HS contribution to 100%.

#### 3.2.2. Molecular structure of mononuclear complex [Pd(L)_2_(Cl)_2_]·2(C_6_H_5_CH_3_) (M)

The mononuclear complex [Pd(L)_2_Cl_2_]·2(C_6_H_5_CH_3_) crystallizes as a solvate containing two toluene molecules in the centrosymmetric space group *P*1̄ of the triclinic system. The asymmetric unit of M consists of half of the Pd complex molecule and one toluene solvent molecule (Fig. 3). The palladium center adopts a square-planar coordination geometry. The Pd–N and Pd–Cl bond lengths, as well as the Cl–Pd–N bond angles (Table 4), are typical of those reported for neutral square-planar Pd(ii) complexes.^76,77^ The Pd–Cl bond length of 2.3148(5) Å and the Cl–Pd–N bond angles of 91.36(6)° and 88.64(6)° are comparable to the corresponding values reported for related square-planar Pd(ii) complexes bearing N-donor ligands, supporting the typical four-coordinate geometry of the Pd(ii) centre in complex M.^12,13^

**Fig. 3:**
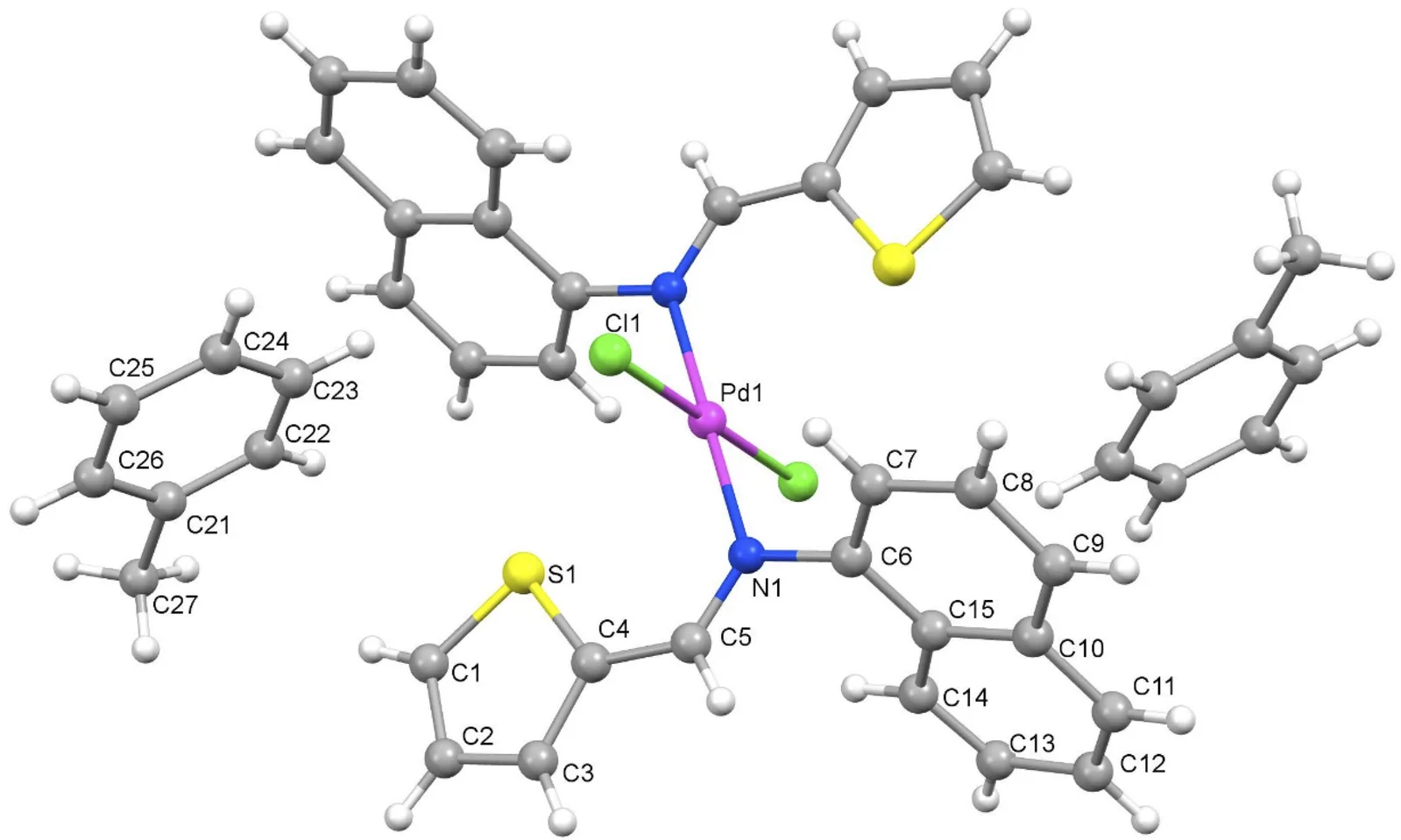
X-ray structure of the mononuclear complex [Pd(L)_2_(Cl)_2_]·2(C_6_H_5_CH_3_) (M) with labelling of atoms in the asymmetric unit.

**Table 4:** Selected geometric parameters around palladium for complex M^a^

	Bond lengths (Å)		Bond angles (°)
Pd1–Cl1	2.3148(5)	Cl1–Pd1–N1	91.36(6)
Pd1–Cl1^*i*^	2.3147(5)	Cl^i^–Pd1–Cl1	180.0
Pd1–N1	2.0221(19)	Cl1–Pd1–N1^*i*^	88.64(6)
Pd1–N1^*i*^	2.0221(19)	N1–Pd1–N1^*i*^	180.0

a Symm code: *i*: 1 − *x*, 1 − *y*, 1 − *z*.

The geometrical parameters of L coordinated to Pd closely resemble those of the free ligand (see the last column of Table 3). Both ligand fragments attached to the central nitrogen atom (N1) remain planar upon coordination, with mean deviations of the constituent atoms from the least-squares plane of 0.008 Å and 0.048 Å for the S1, C1–C5, N1 and N1, C6–C15 fragments, respectively. However, the dihedral angle between the planar fragments of the ligand L after coordination to Pd is 116.4(3)°, considerably larger than that in the free ligand [33.1(4)°]. The conformational change of the ligand is associated with coordination to the central Pd(ii), with the sulfur atom of the ligands lying above or below the deformed square coordination plane of PdCl_2_N_2_. The corresponding S1–Pd1–N1 and S1–Pd1–Cl1 angles are 75.25(6)° and 88.08(2)°, and the S1⋯Pd distance is 3.100(1) Å. In octahedral (six-coordinate) Pd(ii) complexes, the Pd–S axial bond length is approximately 2.80–2.90 Å for extended axial bonds, due to the strong Jahn–Teller effect for the *d*^8^ configuration. It is worth noting, however, that the octahedral geometry is unusual for palladium(ii), which clearly prefers the four-coordinate, square-planar geometry observed here for M.

The crystal structure is composed of corrugated layers of [Pd[C_10_H_7_N=CH(C_4_H_3_S)]Cl_2_] complexes with the solvent molecules inclusions in the voids between layers (Fig. 4). The layers of Pd complexes are interlinked with solvent molecules *via* C–H⋯π and C–H⋯Cl weak interactions. The H⋯π and H⋯Cl distances are 2.925(3) and 2.884(3) Å, respectively.

**Fig. 4:**
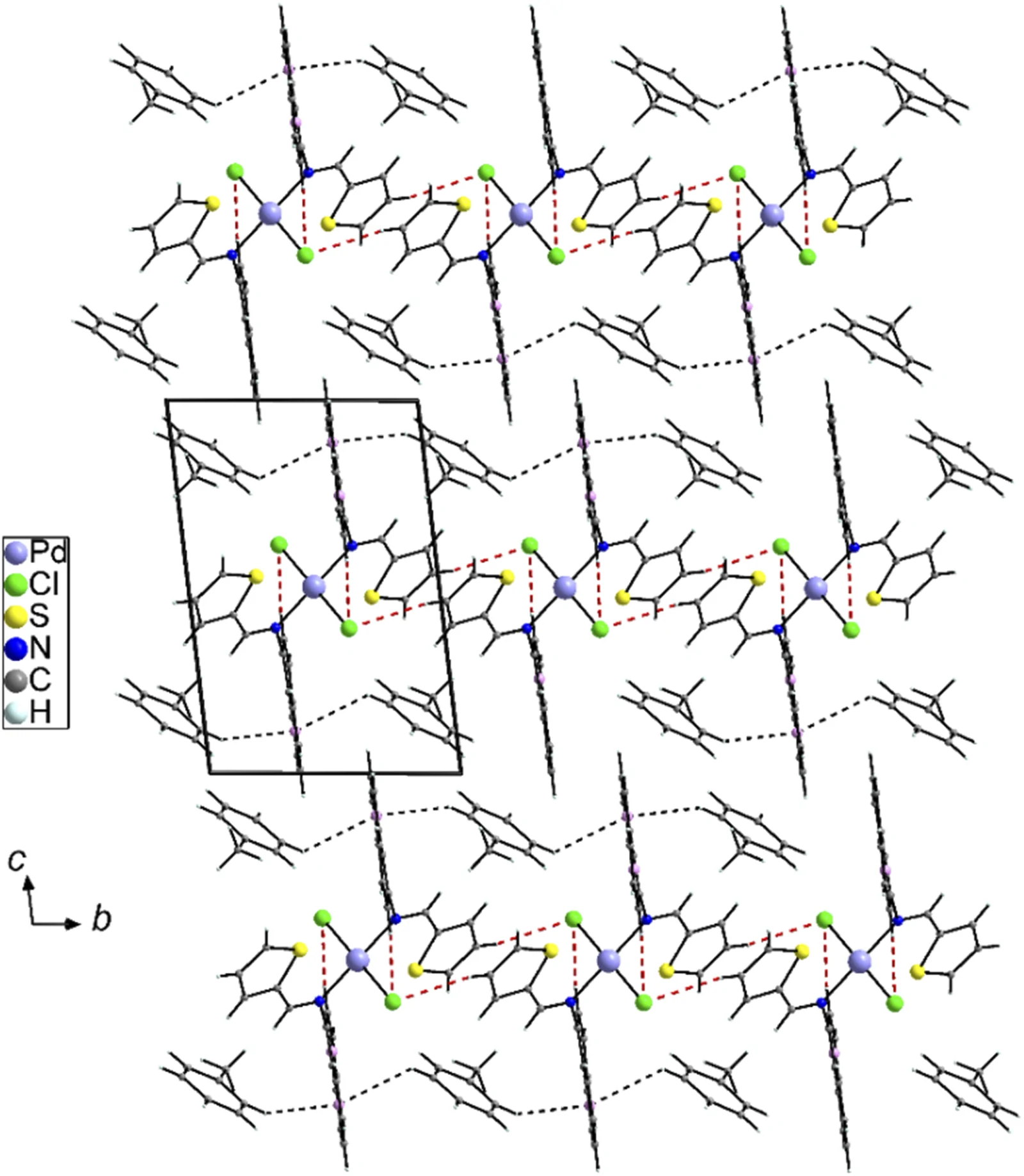
The arrangement of the complex M molecules showing the corrugated layers with the toluene solvent molecules in the voids. The dashed lines show C–H⋯π (black) and C–H⋯Cl (red) weak interactions.

The interactions between the [Pd(L)_2_(Cl)_2_] complex and the toluene solvent molecules were analyzed using HS and the 2DFP plots and the results are shown in Fig. S2 and S3 in SI. However, apart from the previously mentioned weak C–H⋯π and C–H⋯Cl interactions, the main crystal stabilizing interactions are H⋯H dispersion forces and van der Waals interactions, the contribution of which constitutes the major part in HS. The contribution of H⋯H interactions in HS of the [Pd(L)_2_(Cl)_2_] complex molecule is 45.4%, C⋯H/H⋯C interactions constitutes 33.6% and the contributions of H⋯Cl/Cl⋯H and H⋯S/S⋯H interactions are 11.7 and 4.8%, respectively (Fig. S2, SI). The toluene solvent molecules located in the voids of the [Pd(L)_2_(Cl)_2_]·2(C_6_H_5_CH_3_) (M) crystal interact *via* C–H⋯π with naphthalene ring of the coordinated L ligand. However, the main contribution to HS is made by dispersion forces and H⋯C/C⋯H, whose share in HS is over 94% (Fig. S3, SI). Other interactions between solvent toluene molecules and the [Pd(L)_2_(Cl)_2_] complex molecule are H⋯Cl and H⋯S interactions, whose contributions in the HS is 3.3 and 2.1%, respectively.

### 3.3. Catalytic studies

Palladium complexes D^1^–D^4^ were screened as catalysts for the Suzuki–Miyaura cross-coupling of bromobenzene with phenylboronic acid. Complex D^2^, at a 0.47 mol% loading, exhibited the highest catalytic activity, yielding 91% of the biaryl product in 30 minutes at 60 °C (Table 5), and was thus selected for further optimization. Lowering the catalyst loading to 0.2 mol% reduced efficiency, while increasing it to 0.9 mol% offered no significant improvement. Ethanol proved to be the optimal solvent, outperforming other tested solvents, with DMF yielding the lowest conversion (69%). Base screening identified K_2_CO_3_ as the most effective base, surpassing Na_2_CO_3_, KOH, and NaOAc. Temperature studies showed efficient coupling at both 60 °C and 80 °C, but 60 °C was preferred for its operational simplicity and consistent results.

**Table 5:** Optimization of reaction conditions in the Suzuki–Miyaura cross-coupling reaction between bromobenzene and phenylboronic acid^a^

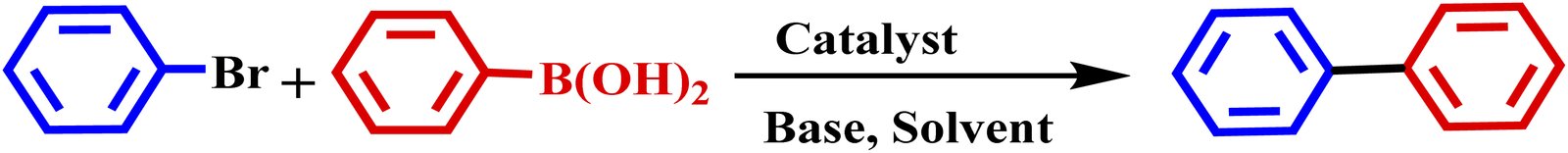						
Entry	Solvent	Base	Catalyst	Loading (mol% Pd)	*T* (°C)	Yield^b^ (%)
**Catalyst screening**						
1	EtOH	K_2_CO_3_	D^1^	0.47	60	82
2	**EtOH**	K_2_CO_3_	D^2^	**0.47**	**60**	**91**
3	EtOH	K_2_CO_3_	D^3^	0.47	60	84
4	EtOH	K_2_CO_3_	D^4^	0.47	60	78
5	EtOH	K_2_CO_3_	D^2^	0.90	60	91
6	EtOH	K_2_CO_3_	D^2^	0.20	60	40

**Solvent study**						
7	DMF	K_2_CO_3_	D^2^	0.47	60	69
8	PEG 400	K_2_CO_3_	D^2^	0.47	60	89
9	Toluene	K_2_CO_3_	D^2^	0.47	60	70
10	**EtOH**	**K**_**2**_ **CO**_**3**_	D^2^	**0.47**	**60**	**91**

**Base study**						
11	EtOH	—	D^2^	0.47	60	NC^c^
12	EtOH	Na_2_CO_3_	D^2^	0.47	60	87
13	**EtOH**	**K**_**2**_ **CO**_**3**_	D^2^	**0.47**	**60**	**91**
14	EtOH	KOH	D^2^	0.47	60	80

**Temperature study**						
15	EtOH	NaOAc	D^2^	0.47	60	78
16	EtOH	K_2_CO_3_	D^2^	0.47	25	46
17	**EtOH**	**K**_**2**_ **CO**_**3**_	D^2^	**0.47**	**60**	**91**
18	EtOH	K_2_CO_3_	D^2^	0.47	80	91

a Reagents and conditions: 0.5 mmol of bromobenzene, 0.6 mmol of phenylboronic acid, 0.75 mmol of base, solvent (2 mL), catalyst, time 10 min, NC (no conversion).

b Isolated yield.

Under these optimized conditions with EtOH as the solvent, K_2_CO_3_ as the base, and 0.47 mol% of catalyst D^2^ at 60 °C, the catalytic system was applied to a range of aryl halides (Table 6). The method proved effective for both aryl bromides and chlorides in reactions with phenylboronic acid. As expected, aryl bromides underwent coupling more readily due to the superior leaving group ability of bromine. Furthermore, the influence of substituents on the aryl halide was examined. Electron-withdrawing groups on the aryl halide enhanced yields (entries 4 and 5, Table 6), while electron-donating groups also afforded good to excellent yields of the corresponding biaryl products.

**Table 6:** Suzuki cross-coupling reaction of substituted aryl halides with phenylboronic acid^a^

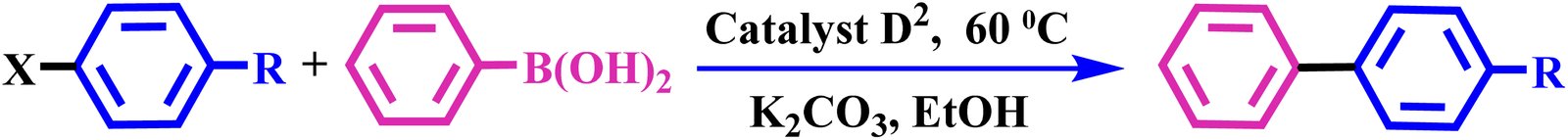						
Entry	R	X	Product	Time (min)	Yield^b^ (%)	TON^c^
1	H	Cl	Ph–Ph	30	76	150
2	CHO	Cl	*p*-OHC–Ph–Ph	40	85	170
3	COOH	Cl	*p*-HOOC-Ph–Ph	30	85	170
4	COOH	Cl	*o*-HOOC-Ph–Ph	10	89	180
5	NO_2_	Br	*p*-O_2_N–Ph–Ph	10	98	200
6	CN	Br	*p*-NC–Ph–Ph	20	98	200
7	COOH	Br	*p*-HOOC-Ph–Ph	15	83	170
8	COCH_3_	Br	*p*-H_3_COC–Ph–Ph	15	90	180
9	CHO	Br	*p*-OHC–Ph–Ph	10	82	160
10	H	Br	Ph–Ph	10	91	180
11	OCH_3_	Br	*p*-H_3_CO–Ph–Ph	40	88	180
12	CH_3_	Br	*p*-H_3_C-Ph–Ph	25	93	190

a Reaction conditions: catalyst D^2^ (0.47 mol%); phenylboronic acid (0.5 mmol); Aryl halides (0.6 mmol); K_2_CO_3_ (0.75 mmol); solvent: EtOH (2 mL); at 60 °C under reflux.

b Detected by TLC.

c TON: turnover number, yield of product/per mol of catalyst.

To demonstrate the catalytic effect of D^2^, the results of the coupling of bromobenzene and phenylboronic acid in the reported method are compared with a set of similar methods available in the literature (Table 7).^17,78–81^ The comparison of the results shows a shorter reaction time, a lower catalyst amount, and better performance for catalyst D^2^. The results obtained show that the use of catalyst D^2^ yields high product yields, has a shorter reaction time, uses ethanol as the reaction solvent, and requires less catalyst. These advantages make the present method a valuable addition to the existing methods available in the literature.

**Table 7:** Comparison of the catalytic activity of complex D^2^ with some reported palladium complexes for the Suzuki cross-coupling

Entry	Catalyst (mol%)	Conditions (solvent, *T*, h)	Yield	Ref.
1	D^2^ – 0.47 mol%	EtOH (2 mL), K_2_CO_3_ (0.75 mmol), reflux ≈ 60 °C, 10 min	98%	This work
2	Pd–iminophosphine	Cs_2_CO_3_/Acetonitrile, 80 °C, 6 h	54%	17
3	[Pd(PPh_3_)(L)](ClO_4_)^a^	K_2_CO_3_/PEG-400,100 °C, 10 h	97%	78
4	[Pd(L_1_–L_6_)_2_]^b^	K_2_CO_3_/DMF,80 °C, 2 h	98%	79
5	[PdCl_2_(P^C)]^c^	K_2_CO_3_/DMF,130 °C, 2 h	97%	80
6	[PdCl_2_(P^N)]^d^	K_2_CO_3_/Toluene, 100 °C, 24 h	80%	81

a L = 2,4-dichloro-6(((2(ethylthio)phenyl)imino)methyl)phenol.

b L_1_ = *N*-(naphthyl)-salicylaldimine, L_2_ = *N*-(naphthyl)-methoxysalicylaldimine, L_3_ = *N*-(biphenyl)-salicylaldimine, L_4_ = *N*-(biphenyl)-methoxysalicylaldimine, L_5_ = *N*-(naphthyl)-2-hydroxy-1-naphthaldimine, L_6_ = *N*-(biphenyl)-2-hydroxy-1-naphthaldimine.

c P^C = 1-([1,1′-biphenyl]-4-yl)-2-(diphenylphosphino){[(diphenylphosphino)]methyl}phosphoranylidene-ethanone ligand (P, C-coordained ligand).

d P^N = 8-(di-*tert*-butylphosphinooxy)quinoline ligand (P, N-coordained ligand).

### 3.4. Cytotoxicity investigations

#### 3.4.1. Assessment of cytotoxic effects

As shown in Fig. 5, the results indicated that the complexes inhibited cell viability and proliferation in a manner dependent on the dosage. Among the tested complexes, D^4^ exhibited the strongest cytotoxic effect against both cell lines. The IC_50_ values for D^4^ were approximately 26.46 µM for A549 cells, and 57.01 µM for HT-29 cells after 24 hours of treatment, leading to a significant reduction in cell viability compared to untreated controls. The A549 cell line demonstrated higher sensitivity to the complexes, with HT-29 cells showing moderate sensitivity, as depicted in Fig. 5. Regarding the cytotoxic efficacy of the synthesized complexes against A549 cancer cells in this study, complex D^4^ demonstrated the most significant activity, with subsequent effects observed in D^2^, D^1^, and D^3^, respectively. Also, regarding the cytotoxic efficacy of the synthesized complexes against HT-29 cancer cells in this study, complex D^4^ demonstrated the most significant activity, with subsequent effects observed in D^2^, D^3^, and D^1^, respectively. To evaluate the selectivity of the synthesized palladium(ii) complexes toward cancer cells, their cytotoxic effects were also assessed on normal fibroblast cells under the same experimental conditions, as shown in Fig. 5. Consistent with the cancer cell lines, cell viability decreased in a dose-dependent manner. However, the magnitude of this decrease was markedly lower than that observed in A549 and HT-29 cells.

**Fig. 5:**
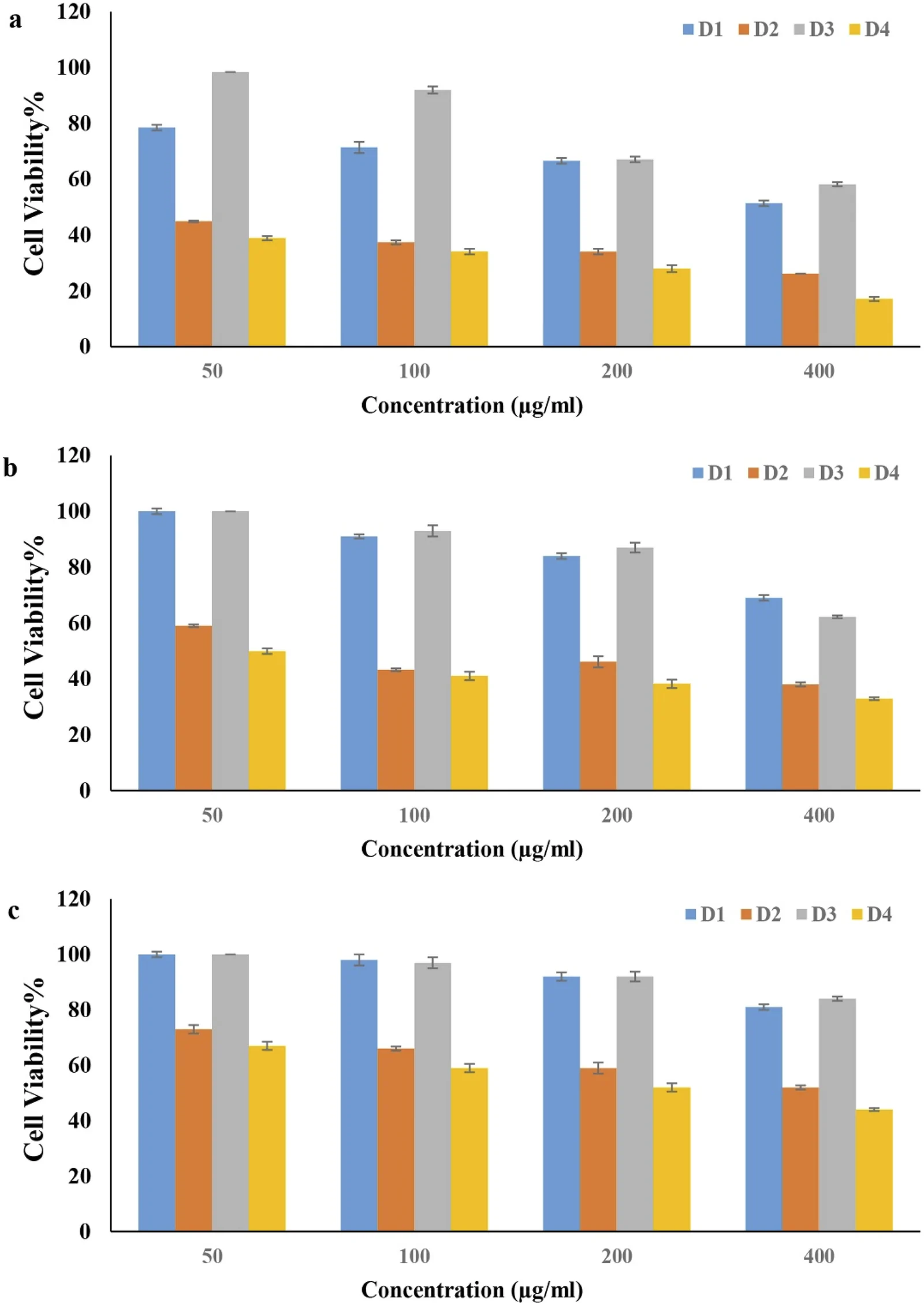
The graph depicts cell viability based on MTT assay results. The absorbance readings, reflecting the proportion of living cells, were obtained from A549 and HT-29 cancer cell lines treated with varying concentrations of the complexes – 50, 100, 200, and 400 µg mL^−1^ – for 24 hours. (a) A549 cells, (b) HT-29 cells, (c) fibroblast cells (*n* = 3).

The nature of these substituents modifies parameters such as lipophilicity, electronic distribution, and reactivity, influencing their interaction with cancer cells. Among the tested complexes, D^4^ demonstrated the most potent cytotoxicity against both A549 and HT-29 cells, with IC_50_ values of approximately 26.46 µM and 57.01 µM, respectively. This suggests that bromine substitution enhances the complexes overall efficacy. Increased lipophilicity imparted by the bromine atom likely facilitates better membrane permeation, allowing D^4^ to reach intracellular targets more effectively. The higher sensitivity of A549 cells might reflect differences in membrane composition, uptake mechanisms, or intrinsic susceptibility compared to HT-29 cells (Fig. 6).

**Fig. 6:**
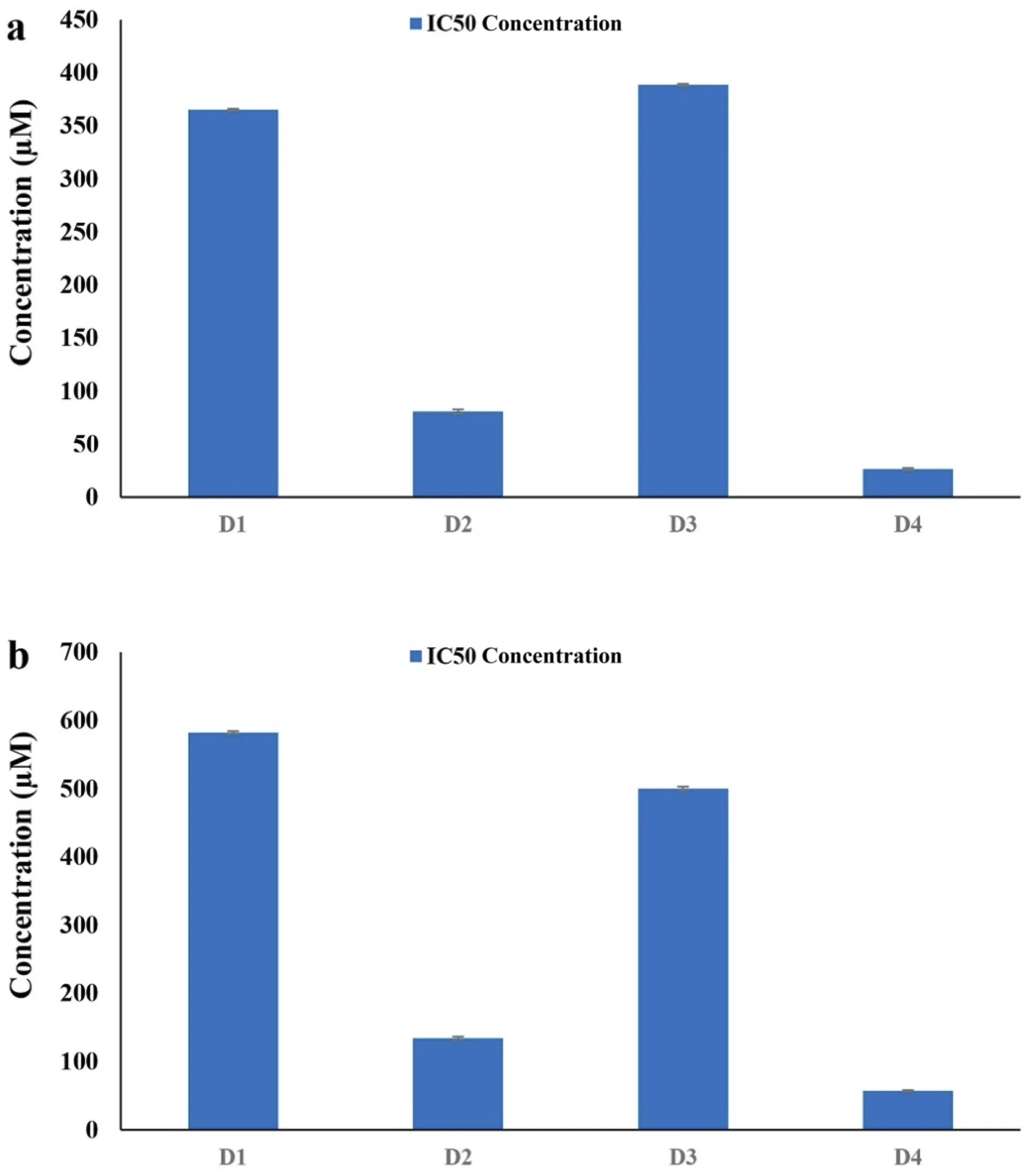
IC_50_ concentration of different complexes in two cell lines: (a) A549 cells, (b) HT-29 cells (*n* = 3).

Complexes (D^1^ and D^2^) showed moderate activity, which may be influenced by their electronic properties – chlorine and nitro groups – potentially affecting their solubility, stability, and interaction with cellular components. Complex D^3^, bearing a methyl group, exhibited the least cytotoxic effect, probably due to its increased hydrophobic character or steric effects that hinder optimal interaction with intracellular targets.

The results indicate that ligand modifications significantly impact the biological activity of these palladium(ii) complexes. Among the series, complex D^4^ exhibits promising anticancer properties, warranting further mechanistic studies, optimization for selectivity, and evaluation of *in vivo* models. Adjusting ligand structures can fine-tune activity and reduce potential side effects, advancing these complexes as prospective chemotherapeutic agents. A comparison of the cytotoxic activity of D^4^ against the A549 and HT-29 cell lines with those of previously reported palladium complexes is presented in Table S5 (SI).^11,82–88^D^4^ exhibited cytotoxic activity comparable to, and in some cases higher than, that of the other palladium complexes. The bioactivity of the palladium complexes may be explained by Tweedy's chelation theory,^89^ according to which chelation considerably reduces the polarity of the central metal ion through partial sharing of its positive charge with the donor atoms of the ligand and possible π-electron delocalization over the chelate ring.

#### 3.4.2. Morphological observations

The morphological assessment revealed that treatment with complex D^4^ caused notable alterations in the appearance of both cell lines, including cell rounding, nuclear condensation, reduction in cell size, and detachment from the culture surfaces, as illustrated in Fig. 7. In contrast, the untreated control cells maintained their typical morphology.

**Fig. 7:**
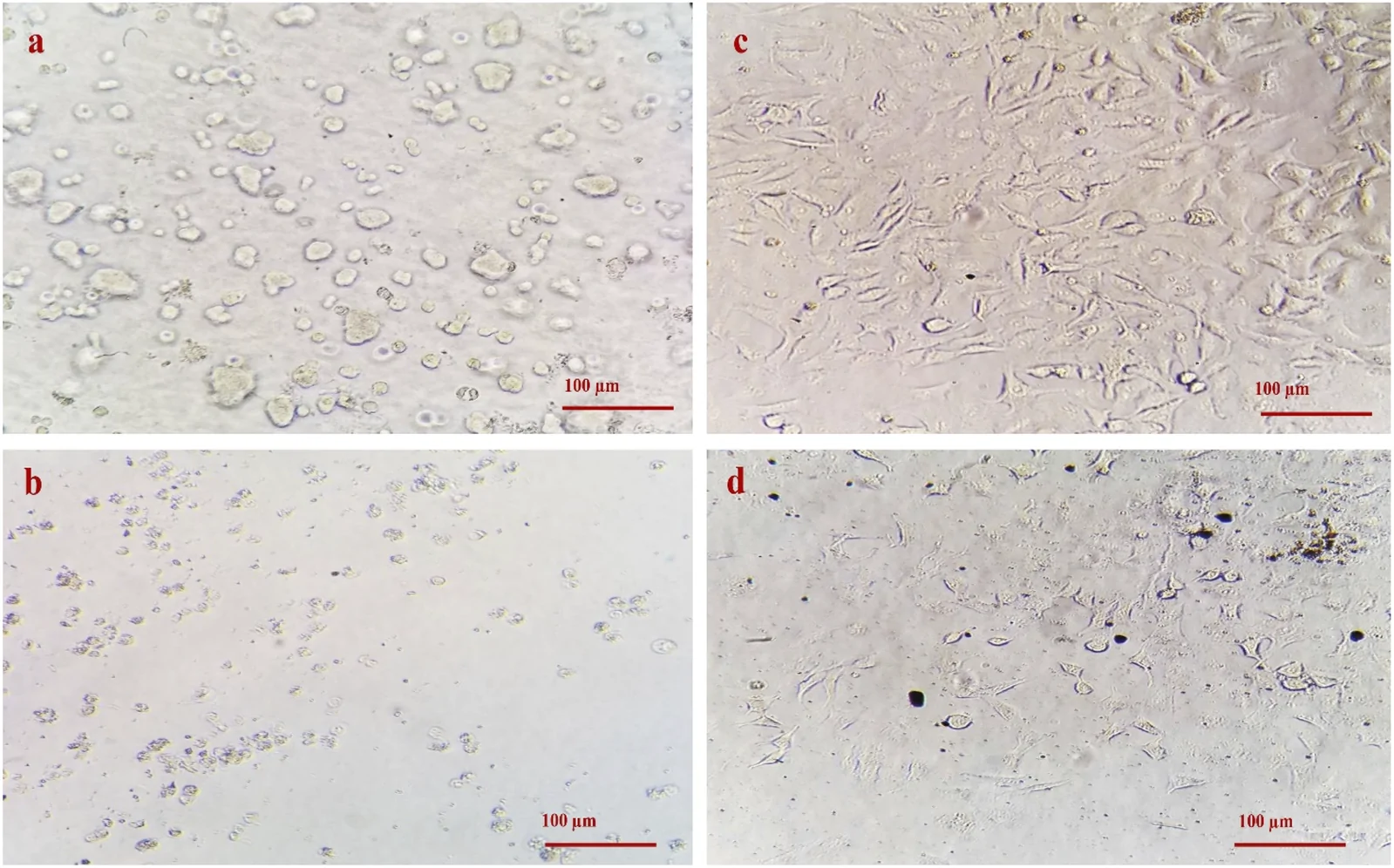
Microscopic examination of cell morphology was conducted to assess the cytotoxic effects of complex D^4^ at its IC_50_ level on A549 and HT-29 cells following 24 hours of treatment. (a) HT-29 control cells, (b) HT-29 cells treated with D^4^, (c) A549 control cells, (d) A549 cells treated with D^4^.

#### 3.4.3. AO/EB staining

Following treatment, A549 and HT-29 cells were subjected to AO/EB staining after 24 hours and examined under a fluorescent microscope using a green filter (500–570 nm). During this analysis, cells undergoing apoptosis exhibited bright orange fluorescence due to membrane damage, whereas viable, non-apoptotic cells appeared green. The control group showed no signs of apoptosis, while apoptotic cells were evident in the treated samples (Fig. 8). These findings suggest that complexes D^4^ induces apoptosis in both A549 and HT-29 cell lines. AO/EB staining further confirmed the induction of apoptosis, with bright orange fluorescence highlighting membrane and nuclear integrity disruption characteristic of programmed cell death. These data support the hypothesis that D^4^ exerts its cytotoxicity primarily through apoptotic pathways, which is desirable for anticancer agents because it minimizes inflammatory consequences compared to necrosis. The superior activity of D^4^ may be ascribed to the bromine's role in increasing lipophilicity and reactivity, facilitating cellular uptake and interaction with molecular targets such as DNA or apoptotic mediators. The presence of a bromine substituent can also influence redox cycling, promoting oxidative stress within cancer cells, and thereby activating apoptosis pathways.

**Fig. 8:**
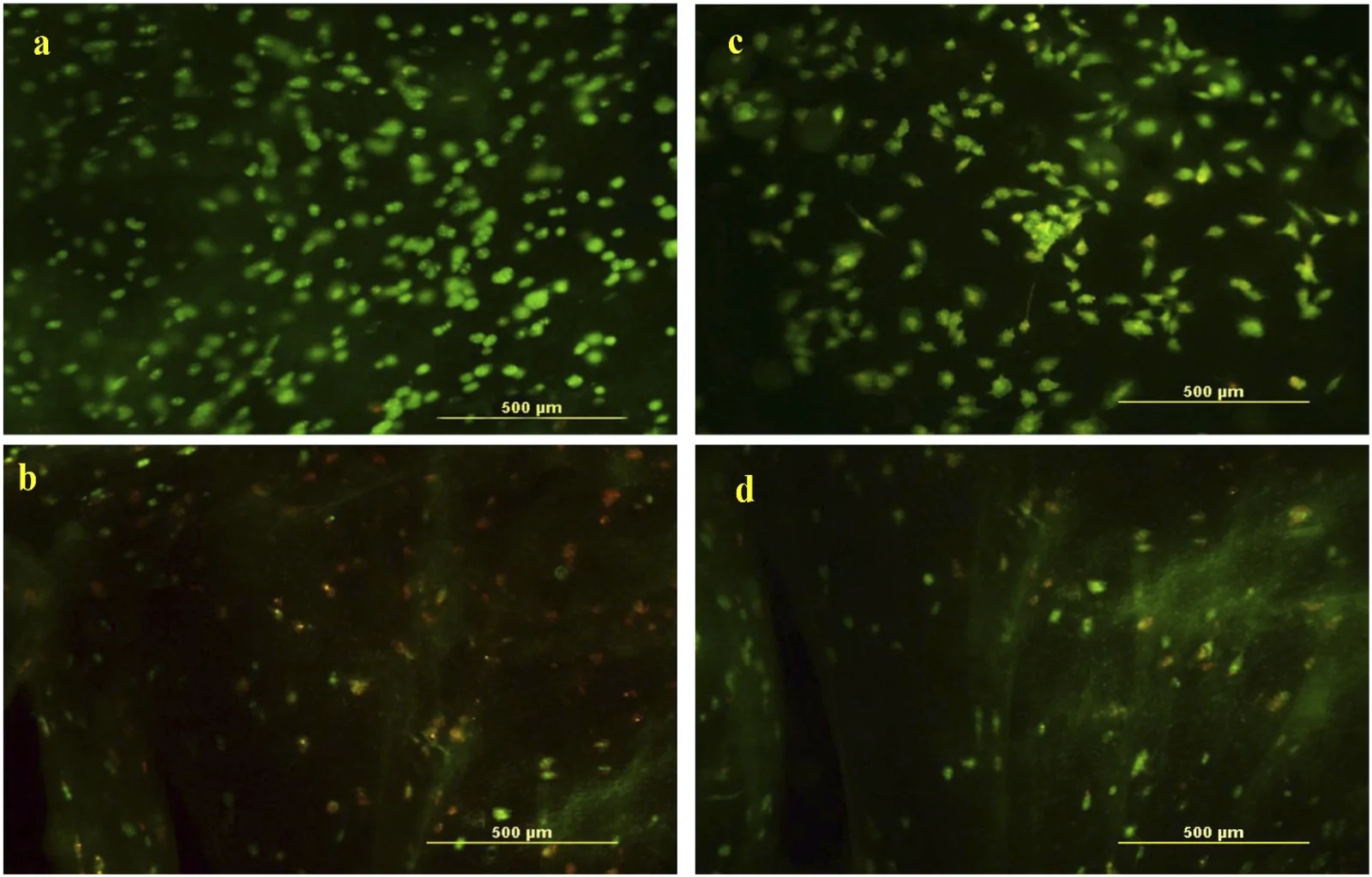
AO/EB staining was used to evaluate the cytotoxic effects of complex D^4^ at its IC_50_ dose on A549 and HT-29 cells after 24 hours of exposure. (a) HT-29 control cells, (b) HT-29 cells treated with D^4^, (c) A549 control cells, (d) A549 cells treated with D^4^.

### 3.5. Molecular docking studies

To rationalize the biological behaviour of L, M and D^1^, molecular docking was performed against two disease-relevant targets. Mpro (PDB ID: 6Y2F), essential for viral polyprotein cleavage, is the principal validated anti-COVID-19 target,^90^ whereas STAT3 – constitutively activated in colorectal and lung carcinomas, the cancer types represented by the cell lines assayed herein – was targeted through its STAT3β/DNA complex (PDB ID: 1BG1).^91,92^ This dual selection allows the docking analysis to address both the antiviral and the anticancer hypotheses raised by the biological data. The atomic binding and interactions of M and D^1^ complexes with its ligand in the presence of proteins (6Y2F) and (1BG1) are shown in Fig. 9 and 10.

**Fig. 9:**
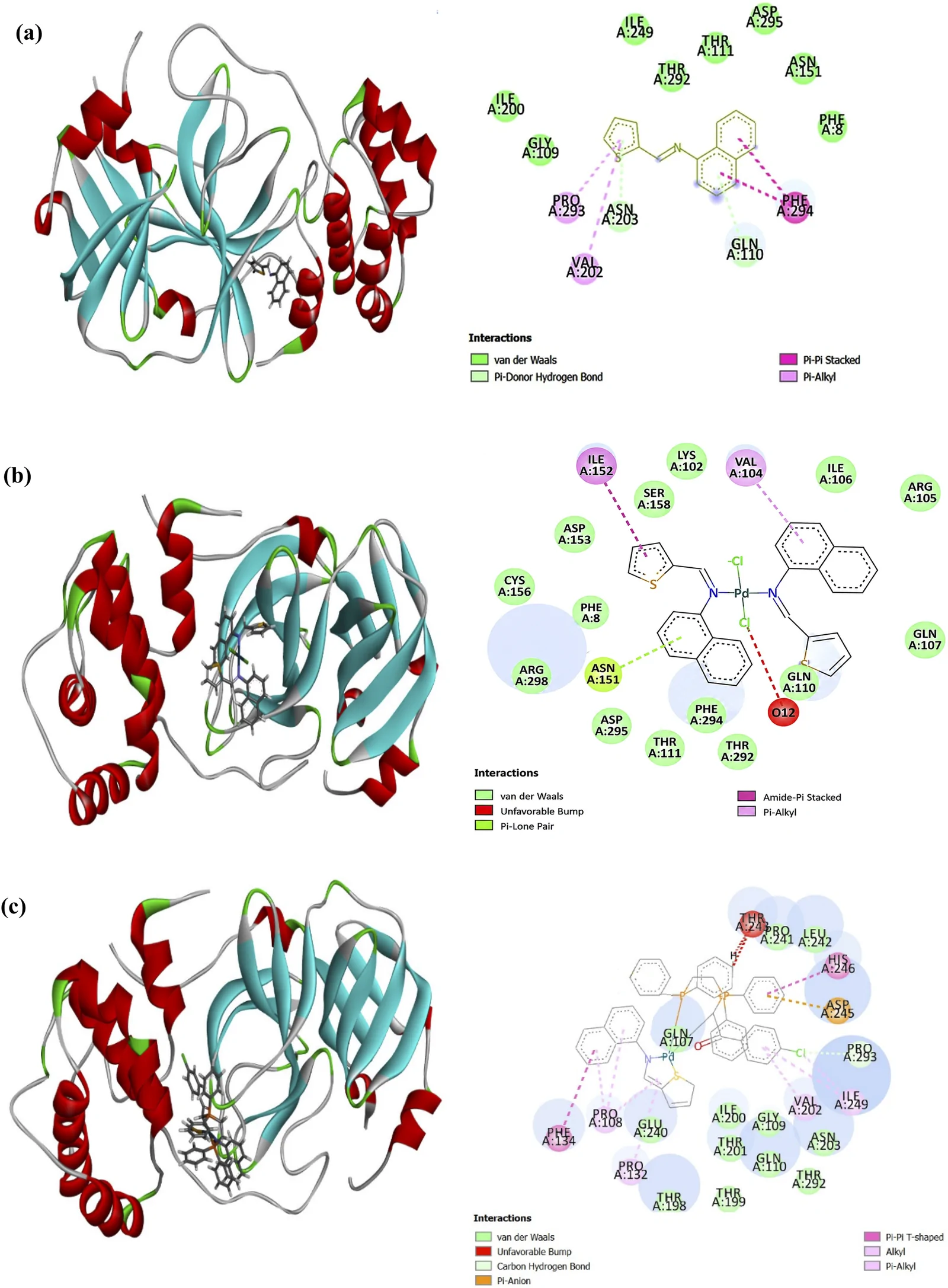
The 3D (left) and 2D (right) interactions of (a) L, (b) complex M and (c) complex D^1^ with 6Y2F.

**Fig. 10:**
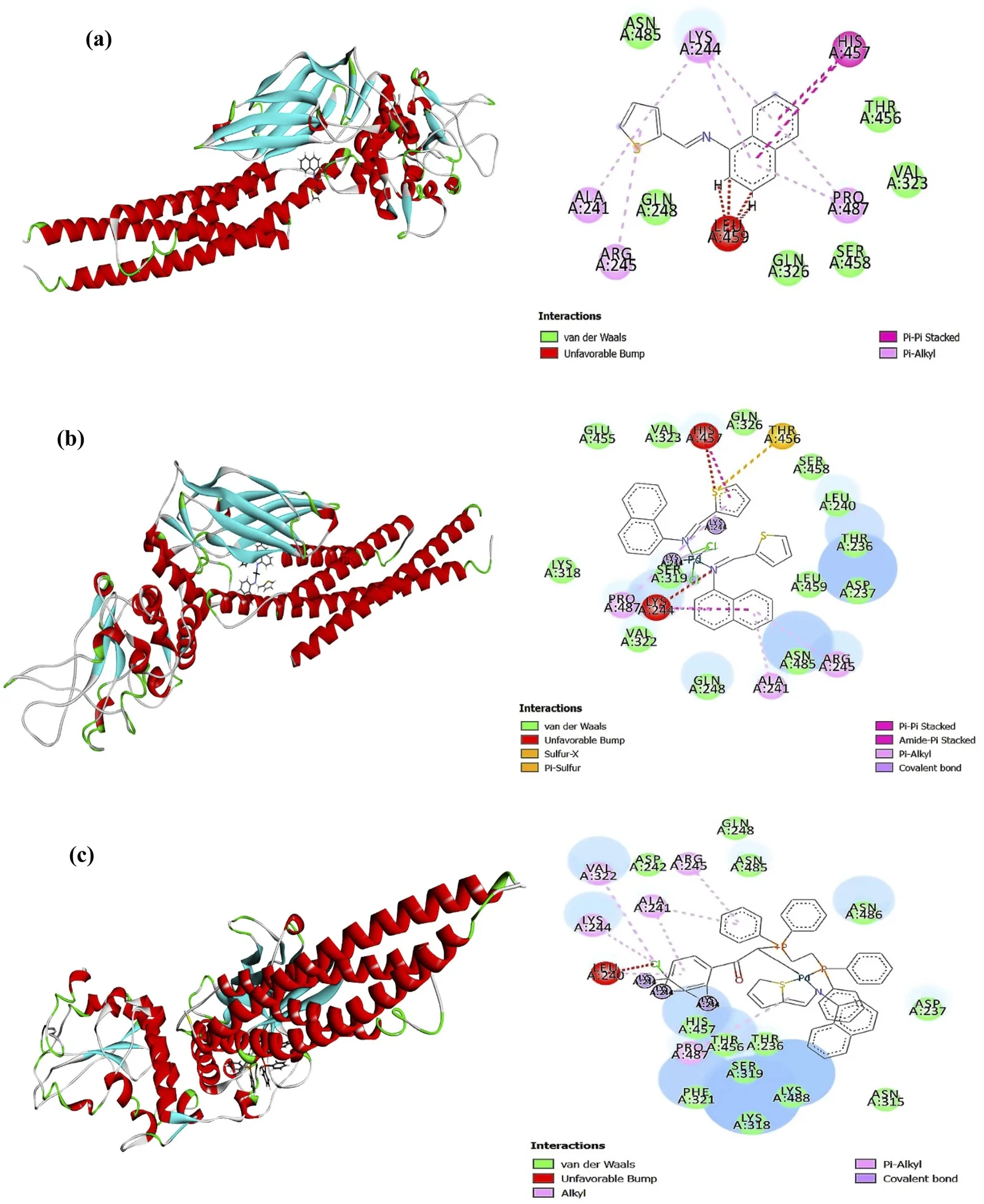
The 3D (left) and 2D (right) interactions of (a) L, (b) complex M and (c) complex D^1^ with 1BG1.

The total energy of the D^1^, M, and L has been calculated to be −141.64, −121.54 and −76.96 kcal mol^−1^ for the (6Y2F) proteins and −100.24, −92.76, −84.19 kcal mol^−1^ for the (1BG1) proteins, respectively. Given that the lower the calculated energy, the greater the efficiency, so D^1^ with a negative energy of −141.64 kcal mol^−1^ in the presence of the (6Y2F) protein shows a better performance than the (1BG1) protein. Analysis of the 2D interaction diagram reveals that the complex is deeply embedded within the binding pocket, surrounded by a diverse array of amino acid residues. The stability of the complex is primarily mediated by several hydrophobic and electrostatic interactions. Specifically, the ligand's aromatic rings are very active, forming multiple π-Alkyl interactions with a group of residues including PHE A:134, PRO A:108, VAL A:202, and ILE A:249. Also, anchoring is provided by a π–π T-shaped interaction with HIS A:246 and a π-anion interaction with ASP A:245, further securing the complex within the site. The thiophene moiety and associated naphthalene group are nestled within a dense hydrophobic environment. This is mediated by an extensive van der Waals network involving residues GLU A:240, PRO A:132, ASN A:203, ILE A:200, and GLN A:110, among others. The widespread light-green contours in the 2D map illustrate the significance of these dispersive forces in stabilizing the ligand's scaffold. In the 1BG1 complex, the binding is characterized by a combination of hydrophobic and electrostatic contacts. The aromatic rings of the ligand establish alkyl and π-alkyl interactions with ARG A:245, ALA A:241, and VAL A:322, facilitating hydrophobic complementarity. Furthermore, the stabilization is reinforced by a π-alkyl contact with PRO A:487. In addition, complex D^1^ formed van der Waals bonds with the amino acids (PRO A:241, A:293), (LEU A:242), (ILE A:249, A:200), (ASN A:203), (GLY A:109), (GLN A:110), (GLU A:240) and (THR A:199, A:201, A:198, A:292) in the protein (6Y2F). In the molecular docking study of the ligand with the active sites of 6Y2F and 1BG1 proteins, various interactions such as van der Waals, π-donor hydrogen bond and π-type interactions were observed. The docking results suggest that the L, M, and D^1^ establish favorable binding interactions within the active sites of Mpro (6Y2F)^90,93^ and STAT3 (1BG1);^91^ inhibition of these proteins would be expected to impair viral replication and cancer-cell proliferation,^94^ respectively. π-Alkyl and π–π T-shaped interactions between the amino and aldehyde ring atoms have been observed with the amino acids valine (VAL A:202), proline (PRO A:108) and phenyl (PHE A:134) for 6Y2F. π-Interactions have been formed with the amino acids (ALA A:241), (LYS A:244), (ARG A:245), (VAL A:322), (LEU A:240) and (PRO A:487) for (1BG1). The results indicate that they possess the ability to effectively penetrate and bind within the active cavity of the receptors. To further contextualize these findings, the binding energies of L, M, and D^1^ were compared with those of related metal complexes and known antiviral/anticancer agents reported against SARS-CoV-2 Mpro and STAT3β/DNA (Table S6, SI). The comparison shows that the studied compounds exhibit binding energies comparable to, and in some instances more favorable than, those previously reported.^51,55,95–97^ Overall, the results indicate favorable binding interactions of L, M, and D^1^ with the investigated targets and provide additional support for their potential as candidates for further biological investigation.

## 4. Conclusion

In this study, mono- and dinuclear thiophene-based palladium(ii) complexes were synthesized from the thiophene-derived Schiff-base ligand L using palladium(ii) chloride and acetate, respectively. Reactivity of dinuclear complex D toward unsymmetrical phosphorus ylides *via* bridge-splitting reactions afforded a new family of mononuclear complexes (D^1^–D^4^). L and its complexes were characterized by spectroscopic and analytical techniques, and the molecular structures of L and M were further confirmed by X-ray crystallography. Complexes D^1^–D^4^ showed good catalytic activity in the Suzuki–Miyaura cross-coupling of phenylboronic acid with aryl halides, with D^2^ giving the best performance. The complexes also showed cytotoxic activity against HT-29 and A549 cancer cell lines, and molecular docking studies indicated favorable interactions of L, M, and D^1^ with the SARS-CoV-2 main protease and STAT3. These findings highlight the potential of thiophene-based Pd(ii) complexes containing phosphorus ylides for further investigation in catalytic and biological applications.

## Conflicts of interest

There are no conflicts to declare.

## Supplementary Material

RA-016-D6RA06822G-s001

RA-016-D6RA06822G-s002

## Data Availability

The data that support the findings of this study are available from the corresponding author upon reasonable request. Supplementary information (SI): additional crystallographic data for complex M (Tables S2–S4). See DOI: https://doi.org/10.1039/d6ra06822g. CCDC 2382487 (L) and 2531664 (M) contain the supplementary crystallographic data for this paper.^98*a*,*b*^
