# Supplementary material for: Mono- and dinuclear thiophene-based palladium(ii) complexes bearing phosphorus ylides: synthesis, catalytic activity, cytotoxicity and molecular docking studies
Source: RSC Adv. 2026 Sep 29;16(45):58585–605. doi: 10.1039/d6ra06822g (PMC13622566; doi:10.1039/d6ra06822g)
Supplement: Supplementary file 1 [file RA-016-D6RA06822G-s001.pdf]

Supplementary Information (SI) for RSC Advances.
This journal is © The Royal Society of Chemistry 2026

Supporting Information

Mono- and dinuclear thiophene-based palladium(II) complexes bearing phosphorus ylides: Synthesis, catalytic activity, cytotoxicity and molecular docking studies

Soraya Sepehrfar[a], Sepideh Samiee[a*], Elham Hoveizi[b], Jan Janczak[c], Vladislav Ignatiev[d] and Paulina Maria Dominiak[d]

[a]*Department of Chemistry, Faculty of Science, Shahid Chamran University of Ahvaz, Ahvaz, Iran*
[b]*Department of Biology, Faculty of Science, Shahid Chamran University of Ahvaz, Ahvaz, Iran*
[c]*Institute of Low Temperature and Structure Research, Polish Academy of Sciences, Okólna 2 str., 50-422 Wroclaw, Poland*
[d]*Biological and Chemical Research Centre, Faculty of Chemistry, University of Warsaw*

---

*Corresponding author. Tel: +986133331042; Fax: +986133337009; *E-mail address*: s.samiee@scu.ac.ir and samiee.sepideh@gmail.com

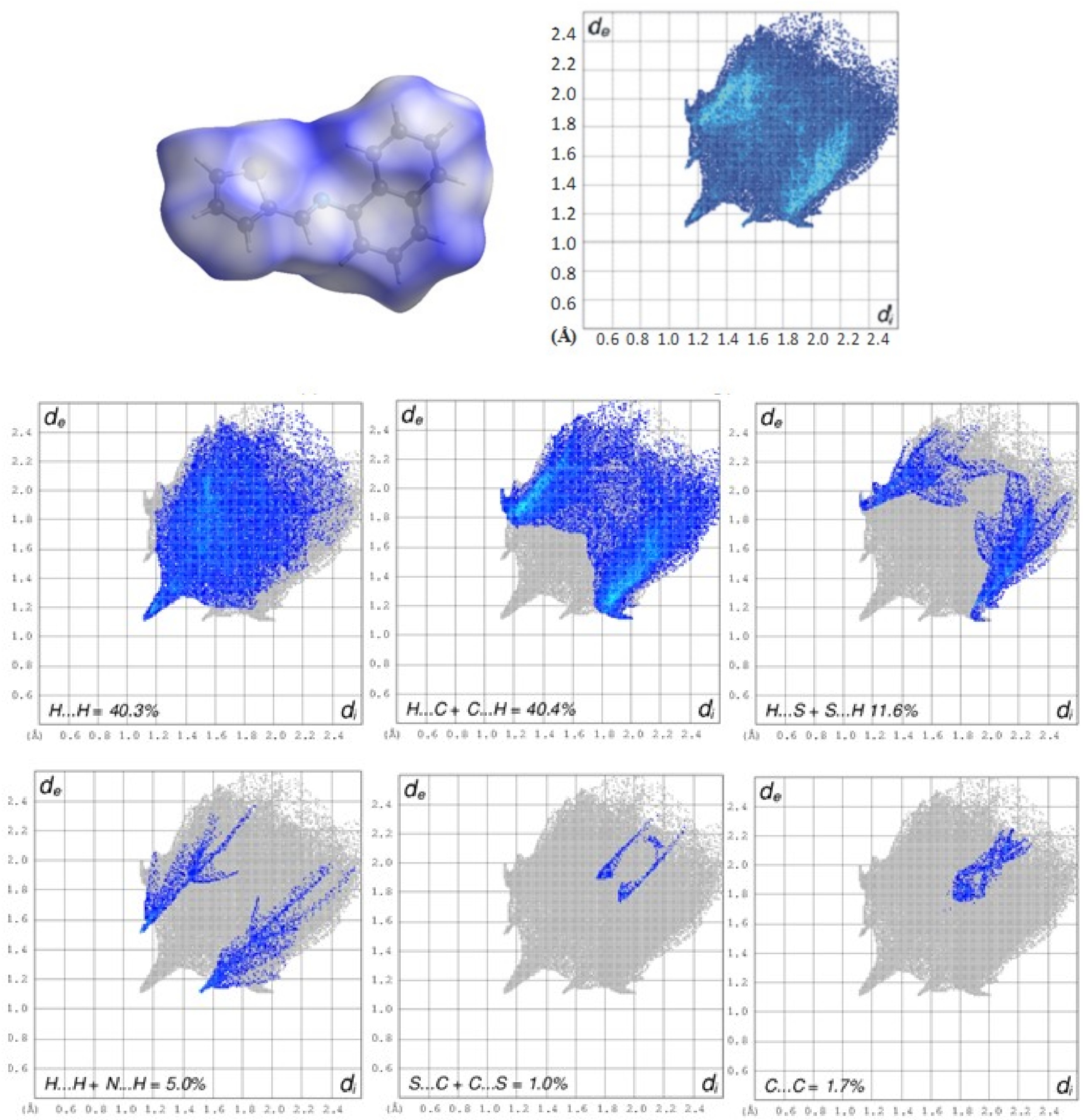

(c)

Figure S1. (a) HS plot and (b) 2DFP plot for the **L** molecule in the crystal, (c) deconvolution of the 2DFP plot into individual types of interactions in the crystal.

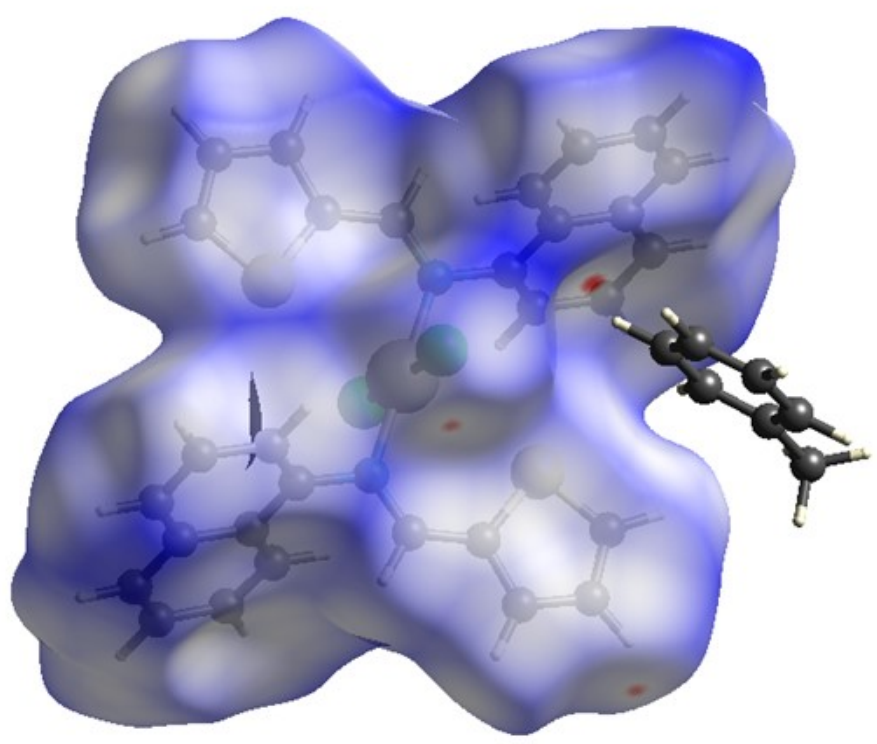

(a)

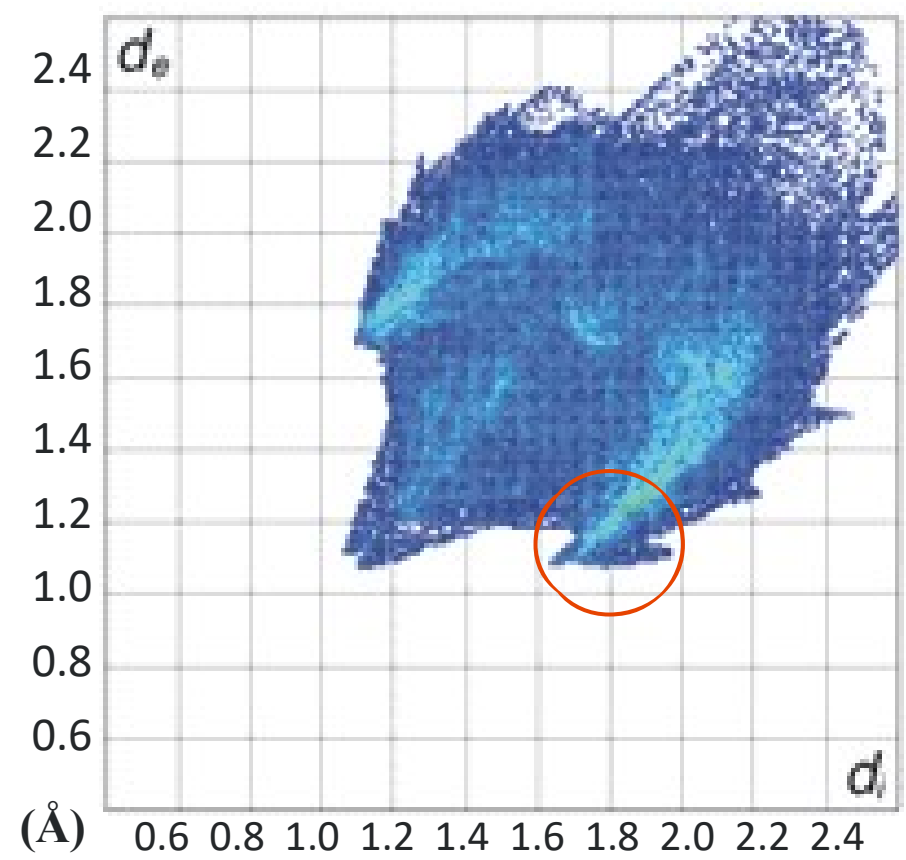

(b)

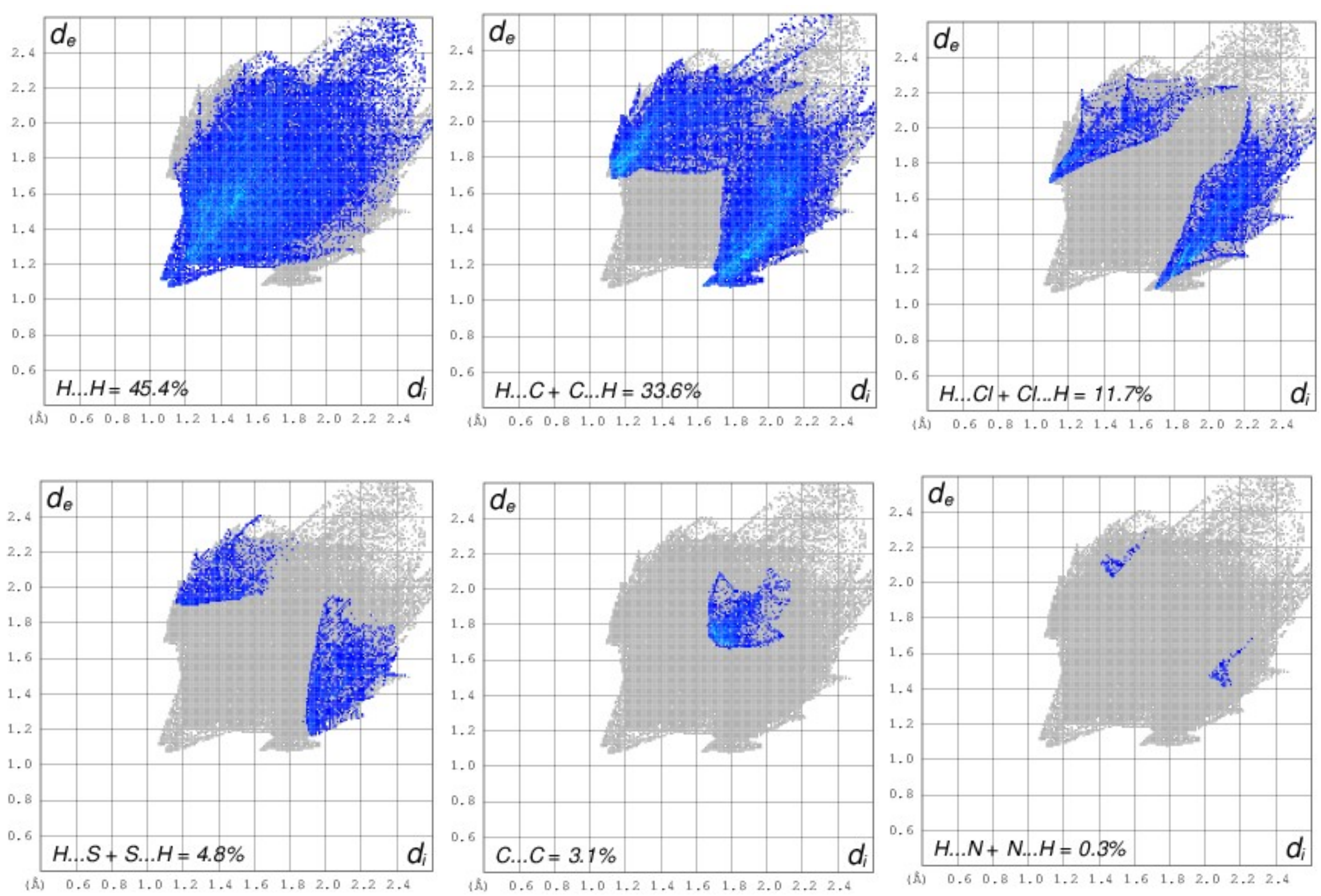

(c)

Figure S2. (a) HS plot and (b) 2DFP plot for the $[Pd(L)_2(Cl)_2]$ complex molecule in the crystal of $[Pd(L)_2(Cl)_2]\cdot 2(C_6H_5CH_3)$ **(M)**, (c) deconvolution of the 2DFP plot into individual types of interactions in the crystal. The red circle indicates the π···H–C interactions between the naphthalene ring of coordinated **L** and solvent toluene molecules.

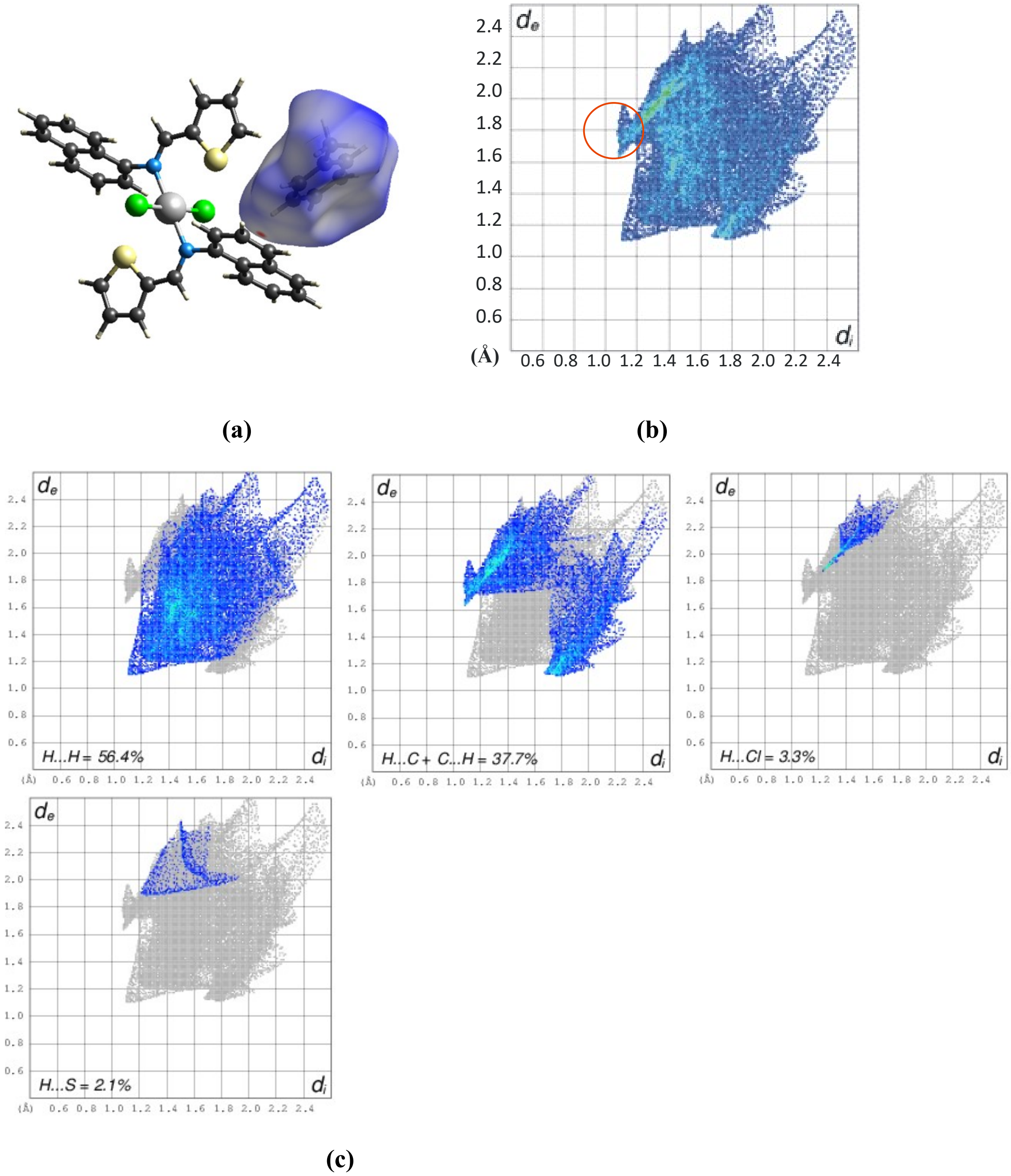

Figure S3. (a) HS plot and (b) 2DFP plot for the toluene molecule in the crystal of $[Pd(L)_2(Cl)_2]2(C_6H_5CH_3)$ (**M**), (c) deconvolution of the 2DFP plot into individual types of interactions in the crystal. The red circle indicates the C–H···π interactions between the solvent toluene molecules and the naphthalene rings.

Table S1. Optimized bond lengths (Å) and bond angles (°) of **L** at the DFT/B3LYP/6-311++G(d,p) level of theory.

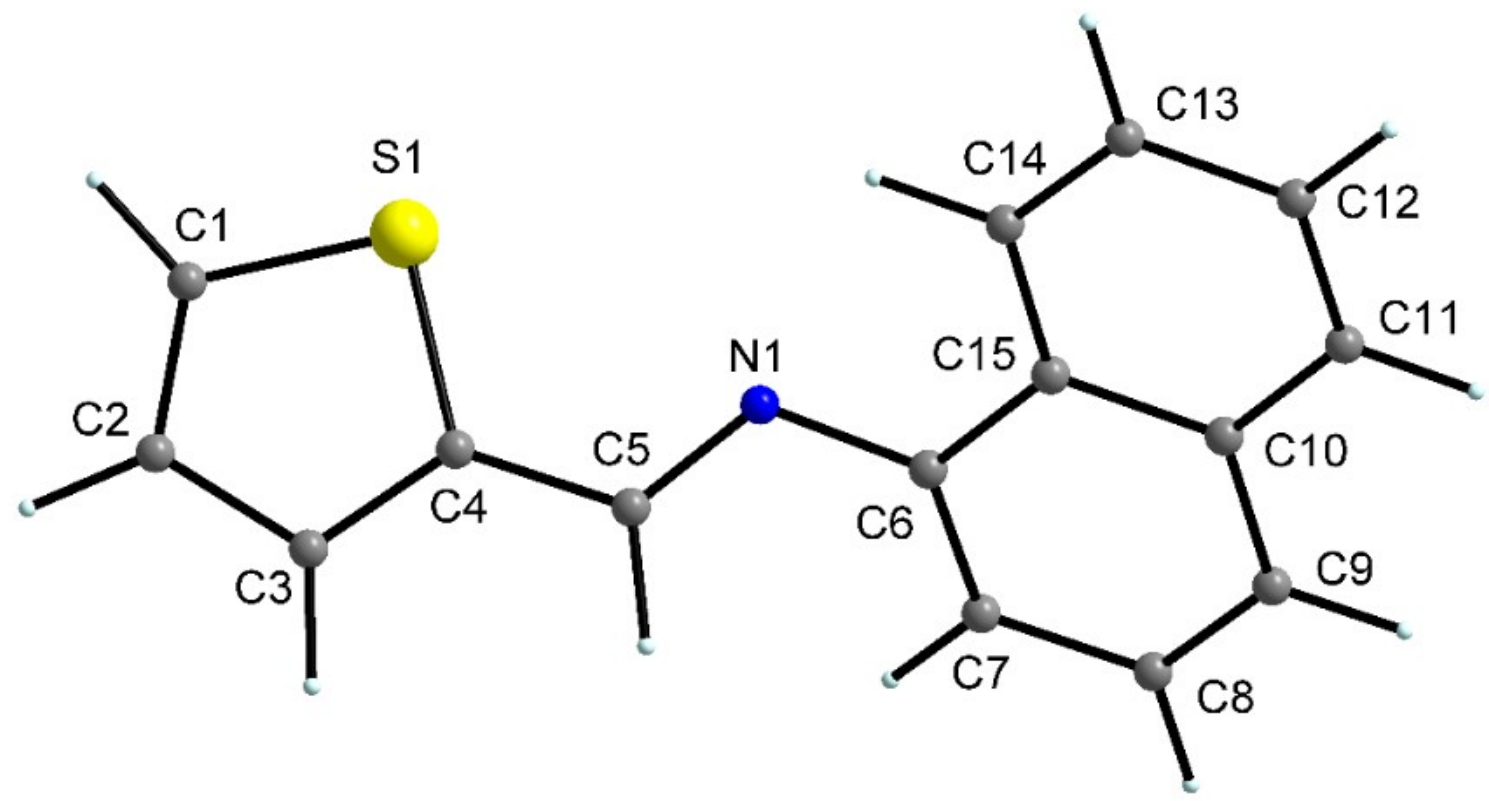

**Bond length (Å)**					
S1–C1	1.729	C1–C2	1.370	C2–C3	1.419
C3–C4	1.379	C4–S1	1.747	C4–C5	1.445
C5–N1	1.279	N1–C6	1.400	C6–C7	1.386
C7–C8	1.411	C8–C9	1.374	C9–C10	1.420
C10–C15	1.430	C10–C11	1.420	C11–C12	1.374
C12–C13	1.414	C13–C14	1.376	C14–C15	1.418
C15–C6	1.435				

C1–H	1.080	C2–H	1.082	C3–H	1.083	C5–H	1.098	C7–H	1.084	C8–H	1.084
C9–H	1.085	C11–H	1.085	C12–H	1.084	C13–H	1.084	C14–H	1.082		

**Bond angles (°)**					
C1–S1–C4	91.12	S1–C1–C2	112.42	C1–C2–C3	112.30
C2–C3–C4	113.23	C3–C4–S1	110.93	C3–C4–C5	126.93
S1–C4–C5	122.15	C4–C5–N1	122.82	C5–N1–C6	120.43
N1–C6–C7	122.46	C6–C7–C8	120.97	C7–C8–C9	120.61
C8–C9–C10	120.43	C9–C10–C11	121.77	C10–C11–C12	120.96
C11–C12–C13	120.14	C12–C13–C14	120.44	C13–C14–C15	120.71
C14–C15–C10	119.00	C14–C15–C6	122.09	C15–C6–N1	117.88

**Torsion angles (°)**					
S1–C1–C2–C3	0.11	C1–C2–C3–C4	0.03	C2–C3–C4–S1	-0.16
C3–C4–S1–C1	0.19	C3–C4–C5–N1	-178.23	C4–C5–N1–C6	-175.79
S1–C4–C5–N1	1.89	C5–N1–C6–C15	-141.03	N1–C6–C15–C14	
C5–N1–C6–C7	42.58	N1–C6–C7–C8	177.90	C6–C7–C8–C9	-0.02
C7–C8–C9–C10	-0.84	C8–C9–C10–C11	-179.57	C9–C10–C11–C12	179.44
C10–C11–C12–C13	-0.16	C11–C12–C13–C14	0.45	C12–C13–C14–C15	-0.29
C13–C14–C15–C6	179.16	C14–C15–C10–C9	-179.29	C15–C15–C6–N1	1.97

The fragments N1–C5–C4–S1–C1–C2–C2–C4 and N1–C6–C7–C8–C9–C10–C11–C12–C13–C14–C15 are planar with the mean deviation of the atoms from the planes of 0.0077 and 0.0074 Å, respectively.

The dihedral angle between the above-mentioned planar fragments is 44.60°.

Table S2. Bond length (Å) for complex **M**.

Atom	Atom	Bond length (Å)	Atom	Atom	Bond length (Å)
Pd1	Cl1	2.3148(5)	C10	C11	1.420(4)
Pd1	Cl1^{i}	2.3147(5)	C14	C13	1.367(3)
Pd1	N1^{i}	2.0221(19)	C14	C15	1.420(3)
Pd1	N1	2.0221(19)	C13	C12	1.411(4)
S1	C4	1.731(2)	C7	C8	1.408(4)
S1	C1	1.709(3)	C2	C1	1.363(4)
N1	C6	1.443(3)	C9	C8	1.367(4)
N1	C5	1.293(3)	C12	C11	1.366(4)
C6	C7	1.376(3)	C21	C26	1.390(4)
C6	C15	1.427(3)	C21	C22	1.399(4)
C5	C4	1.423(3)	C21	C27	1.506(4)
C3	C4	1.398(3)	C26	C25	1.385(4)
C3	C2	1.410(4)	C22	C23	1.386(5)
C10	C9	1.420(4)	C25	C24	1.384(5)
C10	C15	1.425(3)	C23	C24	1.387(5)

Symm Code: i: 1-x, 1-y, 1-z

Table S3. Valence angles (°) for complex **M**.

Atom	Atom	Atom	Angle (°)	Atom	Atom	Atom	Angle (°)
Cl1^{i}	Pd1	Cl1	180.0	C13	C14	C15	120.9(2)
N1^{i}	Pd1	Cl1^{i}	91.36(6)	C14	C13	C12	120.6(2)
N1	Pd1	Cl1^{i}	88.64(6)	C6	C7	C8	120.0(2)
N1	Pd1	Cl1	91.36(6)	C1	C2	C3	112.4(2)
N1^{i}	Pd1	Cl1	88.64(6)	C8	C9	C10	120.6(2)
N1^{i}	Pd1	N1	180.0	C10	C15	C6	117.6(2)
C1	S1	C4	91.15(13)	C14	C15	C6	123.8(2)
C6	N1	Pd1	116.54(14)	C14	C15	C10	118.6(2)
C5	N1	Pd1	126.82(17)	C9	C8	C7	120.6(2)
C5	N1	C6	116.6(2)	C11	C12	C13	120.0(2)
C7	C6	N1	118.9(2)	C12	C11	C10	121.0(2)
C7	C6	C15	121.3(2)	C26	C21	C22	117.6(3)
C15	C6	N1	119.8(2)	C26	C21	C27	120.4(3)
N1	C5	C4	127.6(2)	C22	C21	C27	122.0(3)
C4	C3	C2	112.1(2)	C2	C1	S1	113.2(2)
C5	C4	S1	125.56(18)	C25	C26	C21	121.4(3)
C3	C4	S1	111.11(19)	C23	C22	C21	121.3(3)
C3	C4	C5	123.3(2)	C24	C25	C26	120.3(3)
C9	C10	C15	119.7(2)	C22	C23	C24	120.0(3)
C9	C10	C11	121.4(2)	C25	C24	C23	119.4(3)
C11	C10	C15	118.8(2)				

Symm Code: i: 1-x, 1-y, 1-z

Table S4. Torsion angles (°) for complex **M**.

A	B	C	D	Angle (°)	A	B	C	D	Angle (°)
Pd1	N1	C6	C15	116.9(2)	C2	C3	C4	C5	179.1(2)
Pd1	N1	C5	C4	-1.2(4)	C9	C10	C15	C6	3.2(3)
N1	C6	C7	C8	-176.6(2)	C9	C10	C15	C14	-175.2(2)
N1	C6	C15	C10	174.6(2)	C9	C10	C11	C12	176.0(2)
N1	C6	C15	C14	-7.2(3)	C15	C6	C7	C8	3.3(4)
N1	C5	C4	S1	0.4(4)	C15	C10	C9	C8	1.0(4)
N1	C5	C4	C3	-179.1(2)	C15	C10	C11	C12	-3.0(4)
C6	N1	C5	C4	176.9(2)	C15	C14	C13	C12	-1.0(4)
C6	C7	C8	C9	1.0(4)	C11	C10	C9	C8	-178.1(2)
C5	N1	C6	C7	118.5(2)	C11	C10	C15	C6	-177.8(2)
C5	N1	C6	C15	-61.4(3)	C11	C10	C15	C14	3.9(3)
C3	C2	C1	S1	0.3(3)	C21	C26	C25	C24	-0.5(4)
C4	S1	C1	C2	-0.4(2)	C21	C22	C23	C24	1.0(4)
C4	C3	C2	C1	0.1(3)	C1	S1	C4	C5	-179.0(2)
C10	C9	C8	C7	-3.1(4)	C1	S1	C4	C3	0.46(19)
C14	C13	C12	C11	1.9(4)	C26	C21	C22	C23	-1.8(4)
C13	C14	C15	C6	179.8(2)	C26	C25	C24	C23	-0.4(5)
C13	C14	C15	C10	-1.9(3)	C22	C21	C26	C25	1.5(4)
C13	C12	C11	C10	0.1(4)	C22	C23	C24	C25	0.1(4)
C7	C6	C15	C10	-5.3(3)	C27	C21	C26	C25	-178.2(3)
C7	C6	C15	C14	172.9(2)	C27	C21	C22	C23	177.9(3)

Table S5. Comparison of cytotoxic effects against A549 and HT-29 cell lines between **D⁴** and other complexes reported in the literature

	Cancer Cell line	IC50 (μM)	Ref.
**D⁴**	A549	57.01	This work
$[Pd(Ambim)Cl_2]^a$	A549	23	[82]
$[PdL(PPh_3)]^b$	A549	36	[83]
$[Pd(terpy)Cl]^+$	A549	40.3	[84]
$[Pd(sac)_2(dppm)]^c$	A549	>40	[85]
$[Pd(LH)_2]^d$	A549	36	[11]
**D⁴**	HT-29	26.46	This work
$[Pd_2\{(C,N)–C_6H_4CH_2NH(Et)\}_2(\mu\text{-dppe})(Cl)_2]$	HT-29	7.5	[86]
$[Pd_2\{(C,N)–C_6H_4CH_2NH(Et)\}_2(\mu\text{-dppe})(Br)_2]$	HT-29	5.3	[86]
$[\{Pd(3MAC\text{-}tsc)\}_4]^e$	HT-29	10.5	[87]
$[\{Pd(3MAC\text{-}mtsc)\}_4]^e$	HT-29	10.0	[87]
$[\{Pd(3AC\text{-}etsc)\}_4]^e$	HT-29	12.5	[87]
$[Pd(dppe)(C_6F_5)_2]$	HT-29	>50	[88]

[a] Ambim = 2-aminomethyl benzimidazole
[b] L = N′-(4-(diethylamino)-2-hydroxybenzylidene)furan-2-carbohydrazide
[c] sac = saccharinate
[d] L = N-*p*-chlorophenylthiosemicarbazone
[e] 3-acetyl-chromen-2-one Schiff base ligands [H_2-3MAC-Rtsc] (where R = H [H_2-3MAC-tsc]; CH_3 [H_2-3MAC-mtsc]; C_2H_5 [H_2-3MAC-etsc]

Table S6. Comparative molecular docking binding energies of the present compounds and previously reported metal-based/organic inhibitors against the SARS-CoV-2 main protease (Mpro, PDB: 6Y2F) and the STAT3β/DNA complex (PDB: 1BG1).

	**Total energy of the system (kcalmol^{-1})**		**Ref.**
	**SARS-CoV-2**	**STAT3β/DNA**	
**L**	–76.96	–84.19	This work
**M**	–121.54	–92.76	This work
**D^1**	–141.64	–100.24	This work
$[Co(HL)_2]OAc$[a]	–32.95	–102.54	[95]
$[Ni_2LH_2(CH_3OH)_2](CH_3COO)_2$[b]	–107.13	–75.90	[51]
$[Co^{III}(HL^2)(py)_3]ClO_4$[c]	–82.08	–	[55]
Favipiravir[d]	–44.29	–	[96, 95]
Niclosamide[e]	–	–89.02	[97, 95]

[a] H_2L = (*E*)-5-bromo-2-[2-(hydroxymethyl)phenyliminomethyl]phenol
[b] LH =2-{(*E*)-[(2-hydroxy-1,1-dimethylethyl)imino]methyl}benzene-1,4-diol
[c] HL^2 = (*E*)-*N'*-(3,5-dichloro-2-hydroxybenzylidene)-4-hydroxybenzohydrazide (3,5-dibromo-2-hydroxybenzylidene)
[d] 6-fluoro-3-hydroxy-2-pyrazinecarboxamide, an antiviral drug (as the reference drug)
[e] an FDA-approved anthelminthic drug with antiviral activity (as the reference drug)

References:

11. A. I. Matesanz, E. Jimenez-Faraco, M. C. Ruiz, L. M. Balsa, C. Navarro-Ranninger, I. E. León and A. G. Quiroga, Mononuclear Pd(II) and Pt(II) complexes with an α-N-heterocyclic thiosemicarbazone: cytotoxicity, solution behaviour and interaction versus proven models from biological media, *Inorg. Chem. Front.*, 2018, **5**, 73–83. https://doi.org/10.1039/c7qi00446j

51. S. Parvarinezhad, M. Salehi and M. Kubicki, New dinuclear Schiff base nickel complex: investigation of intermolecular interactions via theoretical calculations, molecular docking studies and ADMET profiling approaches survey, *J. Mol. Struct.*, 2025, **1326**, 141016. https://doi.org/10.1016/j.molstruc.2024.141016

55. S. Parvarinezhad, M. Salehi, M. Kubicki and R. Eshaghi Malekshah, Experimental and theoretical studies of new Co(III) complexes of hydrazide derivatives proposed as multi-target inhibitors of SARS-CoV-2, *Appl. Organomet. Chem.*, 2022, **36**, e6836. https://doi.org/10.1002/aoc.6836

82. I. Mitra, S. Mukherjee, V. P. Reddy B, B. Misini, P. Das, S. Dasgupta, W. Linert and S. C. Moi, Synthesis, biological evaluation, substitution behaviour and DFT study of Pd(II) complexes incorporating benzimidazole derivative, *New J. Chem.*, 2018, **42**, 2574–2589. https://doi.org/10.1039/c7nj05173e

83. T. Thirunavukkarasu, H. A. Sparkes, K. Natarajan and V. Gnanasoundari, ONO pincer-type palladium(II) complexes of heterocyclic hydrazone: synthesis, characterization and biological evaluation, *Appl. Organomet. Chem.*, 2018, **32**, e4403. https://doi.org/10.1002/aoc.4403

84. S. I. Büyükekşi, M. Erkısa, A. Şengül, E. Ulukaya and A. Y. Oral, Structural studies and cytotoxic activity of a new dinuclear coordination compound of palladium(II)-2,2′:6′,2″-terpyridine with rigid dianionic 1,2,4-triazole-3-sulfonate linker, *Appl. Organomet. Chem.*, 2018, **32**. https://doi.org/10.1002/aoc.4406

85. C. Icsel, V. T. Yilmaz, M. Aygun, B. Cevatemre, P. Alper and E. Ulukaya, Palladium(II) and platinum(II) saccharinate complexes with bis(diphenylphosphino)methane/ethane: synthesis, S-phase arrest and ROS-mediated apoptosis in human colon cancer cells, *Dalton Trans.*, 2018, **47**, 11397–11410. https://doi.org/10.1039/c8dt02389a

86. K. Karami, M. Hosseini-Kharat, H. Sadeghi-Aliabadi, J. Lipkowski and M. Mirian, In vitro cytotoxicity studies of palladacyclic complexes containing the symmetric diphosphine bridging ligand: studies of their interactions with DNA and BSA, *Eur. J. Med. Chem.*, 2014, **73**, 8–17. https://doi.org/10.1016/j.ejmech.2013.11.042

87. G. Kalaiarasi, S. Dharani, V. M. Lynch and R. Prabhakaran, Para metallation of 3-acetyl-chromen-2-one Schiff bases in tetranuclear palladacycles: focus on their biomolecular interaction and in vitro cytotoxicity, *Dalton Trans.*, 2019, **48**, 12496–12511. https://doi.org/10.1039/c9dt02663k

88. C. Cullinane, G. B. Deacon, P. R. Drago, A. P. Erven, P. C. Junk, J. Luu, G. Meyer, S. Schmitz, I. Ott and J. Schur, Synthesis and antiproliferative activity of a series of new platinum and palladium diphosphane complexes, *Dalton Trans.*, 2018, **47**, 1918–1932. https://doi.org/10.1039/c7dt04615d

95. M. Salehi, S. Parvarinezhad, P. Roozbahani, R. Eshaghi Malekshah, S. Sepehrfar, M. Kubicki, A. Tarahhomi and G. Dutkiewicz, Synthesis, structural elucidation, and in silico studies of Co(III) and Cu(II) complexes containing from 2-aminobenzyl alcohol: comprehensive characterization, DFT, Hirshfeld surface analysis and molecular docking, *Results Chem.*, 2026, **29**, 103714. https://doi.org/10.1016/j.rechem.2026.103714

96. Y. Furuta, B. B. Gowen, K. Takahashi, K. Shiraki, D. F. Smee and D. L. Barnard, Favipiravir (T-705), a novel viral RNA polymerase inhibitor, *Antiviral Res.*, 2013, **100**, 446–454. https://doi.org/10.1016/j.antiviral.2013.09.015

97. J. Xu, P.-Y. Shi, H. Li and J. Zhou, Broad spectrum antiviral agent niclosamide and its therapeutic potential, *ACS Infect. Dis.*, 2020, **6**, 909. https://doi.org/10.1021/acsinfecdis.0c00052
